# Inherent seizure susceptibility in patients with antihistamine-induced acute symptomatic seizure: a resting-state EEG analysis

**DOI:** 10.1038/s41598-023-36415-7

**Published:** 2023-06-05

**Authors:** Hayom Kim, In-Nea Wang, Jun-Su Park, Keun-Tae Kim, Jooheon Kong, Jung Bin Kim, Dong-Joo Kim

**Affiliations:** 1 Department of Neurology, Korea University Anam Hospital, Korea University College of Medicine, Seoul, Republic of Korea; 2 Department of Brain and Cognitive Engineering, Korea University, Seoul, Republic of Korea; 3 NeuroTx, Co., Ltd., Seoul, Republic of Korea; 4 Department of Artificial Intelligence, Korea University, Seoul, Republic of Korea

**Keywords:** Computational biology and bioinformatics, Neurology

## Abstract

We compared neural activities and network properties between the antihistamine-induced seizures (AIS) and seizure-free groups, with the hypothesis that patients with AIS might have inherently increased neural activities and network properties that are easily synchronized. Resting-state electroencephalography (EEG) data were collected from 27 AIS patients and 30 healthy adults who had never had a seizure. Power spectral density analysis was used to compare neural activities in each localized region. Functional connectivity (FC) was measured using coherence, and graph theoretical analyses were performed to compare network properties between the groups. Machine learning algorithms were applied using measurements found to be different between the groups in the EEG analyses as input features. Compared with the seizure-free group, the AIS group showed a higher spectral power in the entire regions of the delta, theta, and beta bands, as well as in the frontal areas of the alpha band. The AIS group had a higher overall FC strength, as well as a shorter characteristic path length in the theta band and higher global efficiency, local efficiency, and clustering coefficient in the beta band than the seizure-free group. The Support Vector Machine, k-Nearest Neighbor, and Random Forest models distinguished the AIS group from the seizure-free group with a high accuracy of more than 99%. The AIS group had seizure susceptibility considering both regional neural activities and functional network properties. Our findings provide insights into the underlying pathophysiological mechanisms of AIS and may be useful for the differential diagnosis of new-onset seizures in the clinical setting.

## Introduction

An acute symptomatic seizure (ASS) occurs at the time of a systemic insult or in close proximity to a documented brain insult, which may include metabolic, toxic, structural, infectious, or inflammatory injuries^1^. In general, ASSs can be self-limited by avoiding exposure to the sources of brain injury; therefore, the mainstay of ASS management is the identification and correction of predisposing conditions to seizures rather than treatment with antiseizure medications^1,2^. Since ASSs account for approximately 40% of all new-onset seizures^3^, the new-onset seizure must be distinguished as an ASS or an epileptic seizure during the initial evaluation. Among the proximate causes of ASS, a variety of medications, including antihistamines, stimulants, antidepressants, and antibiotics, have been reported as offending agents for seizure development^4,5^.

A recent observational study reported that antihistamines accounted for the greatest proportion of drug-induced ASSs^6^. Because antihistamines are commonly used as over-the-counter medications for treating the common cold, allergic rhinitis, dermatitis, and urticaria, as well as for relieving symptoms of coronavirus infectious disease 2019 around the world, antihistamines should be investigated as a potential cause of new-onset ASSs. On the other hand, considering that the incidence of antihistamine-induced ASSs (AISs) is relatively low despite the extremely high amount of antihistamine use, susceptibility to developing seizures from antihistamine use plausibly varies from person to person. Although reliable markers for seizure susceptibility in patients with AIS can provide important information for differential diagnosis of new-onset seizures and development of management strategies, no established evaluation tools for seizure susceptibility in response to antihistamine use are available.

Several lines of evidence suggest that antihistamines can induce neuronal excitability in the ventral tegmental area, substantia nigra, and ventromedial hypothalamus by acting on histamine receptors and enhance hippocampal CA1 synaptic excitation, leading to increase in seizure susceptibility and severity^7–9^. Considering that antihistamines tend to induce seizures by increasing electrical activity in specific brain regions and by enhancing the synaptic transmission of neuronal excitability^7,9^, quantitative electroencephalography (EEG) analyses of both neuronal activities in each brain region and large-scale network properties for the transmission of excitability may be optimal approaches for quantitatively evaluating seizure susceptibility after antihistamine use and for the diagnosis of AIS.

Herein, we aimed to compare the neuronal activity in each localized brain region and functional network properties between patients with AIS and healthy adults who never had a seizure (seizure-free group) during the resting-state using power spectral density (PSD) and graph theoretical network analyses with functional connectivity (FC) measurements, respectively. In general, a seizure is generated from increased neural activity in a specific brain region and then synchronized with other regions. Therefore, the vulnerability that can cause seizures may be identified using the measurements. In addition, if there were differences in the indices from quantitative resting-state EEG analyses, we used machine learning algorithms to assess the applicability of EEG measurements representing seizure susceptibility as markers for the differential diagnosis between the AIS and seizure-free groups. We hypothesized that the AIS group, relative to the seizure-free group, might have inherent EEG properties that predispose it to seizures, and the distinguishing patterns could be revealed even during seizure-free resting-state. We also hypothesized that machine learning models using EEG indices reflecting seizure susceptibility, which was derived from data acquired during seizure-free resting-state, can discriminate between the AIS and seizure-free groups with high accuracy.

## Methods and materials

### Participants

This study was based on analyses of long-term video-EEG monitoring data acquired from January 2018 to December 2021 in Korea University Anam Hospital and was an extension of our previous work^6^. We selected data from the database, an archive of EEG raw data acquired at the Clinical Neurophysiology Laboratory of Korea University Anam Hospital, of patients who had completed comprehensive tests for the differential diagnosis of new-onset seizures. Serologic laboratory tests, autonomic function tests, brain magnetic resonance imaging with angiography, electrocardiography, and echocardiography were all performed on all patients. Twenty-seven patients with seizures that had a close temporal relationship with the administration of an antihistamine agent, no recurrences after discontinuation of the drug, and who met the criteria for the World Health Organization-Uppsala Monitoring Centre causality classifications of "certain" and "probable" were assigned to the AIS group^10^. For the seizure-free control group, EEG data from 30 age- and gender-matched healthy adults who had never experienced seizures were used.

### EEG acquisition and preprocessing

The EEG tests lasted 30 min and were conducted with a 32-channel recording system (COMET plus; Grass Technologies Inc., West Warwick, RI, USA) and 19 scalp electrodes (Fp1, F7, T3, T5, O1, Fp2, F8, T4, T6, O2, F3, C3, P3, F4, C4, P4, Fz, Cz, and Pz) placed according to the international 10–20 system. The EEG data were sampled at 200 Hz and the bandpass filter was set between 0.1 and 70 Hz. Two board-certified neurologists carefully reviewed and selected ten non-consecutive resting-state 2-s epochs for each participant based on the following criteria: (1) the presence of continuous physiological alpha activity with maximum voltage in the posterior regions; (2) the absence of artifacts, epileptiform discharges, and other nonstationary elements; and (3) the absence of drowsiness or arousal patterns. After that, the epochs were bandpass filtered into the following frequency bands: delta (0.5–4 Hz), theta (4–8 Hz), alpha (8–13 Hz), beta (13–30 Hz), and gamma (30–50 Hz). Following analyses were carried out separately for each band.

### PSD analysis

The spectral density approach is used to extract signal information from a stochastic process that depicts the power distribution in the spectral domain^11^. A fast Fourier transform can be used to estimate the frequency spectrum *X*(*f*) of the EEG, and the power spectrum ${P}_{x}\left(f\right)$ is calculated as ${P}_{x}\left(f\right)=\frac{1}{N}{\left|X\left(f\right)\right|}^{2}$, where *N* is the number of data points. The absolute value of the PSD may express different information depending on individual differences and age^12^; however, the relative PSD, which represents the relative value of the PSD of a specific frequency band compared with the entire frequency range of the signal, can provide reliable information regardless of individual differences and age. Therefore, in this study, the relative power $PSD\left(h\right)$ was calculated for each frequency band as follows:

$$PSD\left(h\right)=\frac{{\int }_{{f}_{l}}^{{f}_{h}}{P}_{x}\left(f\right)df}{\int {P}_{x}\left(f\right)df},$$

where $h$ denotes each frequency band of the EEG signal and ${f}_{h}$ and ${f}_{l}$ denote upper and lower frequencies, respectively. PSD estimation was implemented through the Welch's approach using the Python package^13^. Between-group comparisons of the PSD in each channel between the AIS and seizure-free groups were performed using independent *t*-tests. Statistical significance was set at P < 0.05.

### FC and graph theoretical analyses

Resting-state FC was measured using coherence, which reflects the level of functional signal communication between different brain regions^14^. Coherence values were simultaenously calculated within the same ten segments of 2-s epochs in each frequency band in the FC analysis. Coherence is defined as ${COH}_{xy}={k}_{xy}^{2}\left(f\right)={\left|{K}_{xy}\left(F\right)\right|}^{2}=\frac{{\left|{S}_{xy}\left(F\right)\right|}^{2}}{{S}_{xx}\left(f\right){S}_{yy}\left(f\right)}$, where *x* and *y* correspond to the measured EEG signal in each channel, and *S*_*xx*_(*f*) and *S*_*yy*_(*f*) are the auto-spectral densities of *x* and *y*, respectively, and *S*_*xy*_(*f*) is the cross-spectral density between *x* and *y*. *K* and *|S|* denote the coherence function and modulus of *S*, respectively. The coherence value ranges between 0 and 1, with 0 denoting no statistical relationship and 1 denoting full coherence^14^. Coherence was implemented using the MNE-Python library^15^. To avoid the arbitrariness of threshold selection and preserve the continuous nature of the correlated information, network properties were characterized using a weighted undirected network model of graph-theoretic analysis^16^. Global graph measures representing the degree of integration and segregation properties, which quantitatively reflect the synchronizability in perspective of large-scale brain network were selected for analysis. Graph measures (average degree, average strength, radius, diameter, characteristic path length, global efficiency, local efficiency, clustering coefficient, transitivity, modularity, assortativity, and small-worldness) were computed using the Brain Connectivity Toolbox (http://www.brain-connectivity-toolbox.net)^16^ and the BRAPH toolbox (http://braph.org)^17^ using MATLAB R2022b (MathWorks, Natick, MA, USA). With 1000 permutations, non-parametric tests were used to compare the graph measures between the AIS and seizure-free groups. Statistical significance was set at P < 0.05 and corrected for multiple comparisons using the false discovery rate (FDR).

### Application of machine learning classifiers

A total of 621 measurements (68 PSD, 559 FC, and 4 graph measures) exhibited statistically significant differences between the AIS and seizure-free groups and were selected as input features for the machine learning algorithms. The machine learning classifiers using the EEG measurements were applied to classify the output into one of the two groups. Our previous studies have provided descriptions of feature selection methods for the application of machine learning algorithms^18–20^. This study used the scikit-learn module for Python to implement various machine learning classification algorithms to differentiate the AIS and seizure-free groups, such as kernel Support Vector Machine (kernel SVM)^21^, k-Nearest Neighbor (k-NN)^22^, Random Forest (RF)^23^, Extreme Gradient Boosting (XGBoost)^24^, and Light Gradient Boosting Machine (Light BMG)^25,26^. The classification of the machine learning methods was validated using a random sampling algorithm with class-balanced fivefold cross-validation. Specifically, the entire dataset was randomly split into 80% for the training set and 20% for the testing set. The models were learned using optimal parameters selected through grid search analysis. The performance of each classifier was evaluated using a confusion matrix containing the parameters of precision, recall, accuracy, and F1 score as follows:

$$Precision=\frac{TP}{\left(TP+FP\right)}$$

$$Recall=\frac{TP}{\left(TP+FN\right)}$$

$$Accuracy=\frac{\left(TP+TN\right)}{\left(TP+TN+FP+FN\right)}$$

$$F1score=\frac{2\times precision\times recall}{precision+recall},$$

where TP, FP, TN, and FN represent the true positives, false positives, true negatives, and false negatives, respectively. The recall and specificity were used to generate a receiver operating characteristic (ROC) curve. The area under the ROC curve (AUC) was also calculated.

### Ethics approval and consent to participate

All procedures performed in studies involving human participants were in accordance with the ethical standards of the institutional research committees and with the 1964 Helsinki Declaration and its later amendments or comparable ethical standards. Study was approved by the Ethics Committee of Korea University Anam Hospital (No. 2021AN0307). Informed consent was obtained from all individual participants included in the study.

## Results

### Demographic and clinical characteristics

Demographic and clinical characteristics of the AIS group are presented in Table 1. The age (48.19 ± 22.66 vs. 47.50 ± 15.68, P = 0.90) and sex (37.04% vs. 56.67% of women, P = 0.14) were not different between the AIS and seizure-free groups. All the patients with AIS had experienced only one seizure and took antihistamines at the therapeutic dosage. Both first- and second-generation antihistamines caused seizures. EEG data were acquired at least 24 h after the seizure onset. Long-term video-EEG monitoring visual inspection results, as well as serologic laboratory tests, autonomic function tests, brain magnetic resonance imaging with angiography, electrocardiography, and echocardiography, were all unremarkable. The mean time interval between administering the first antihistamine dose and seizure development was 3.52 days.

**Table 1:** Demographic and clinical characteristics.

	AIS (*n* = 27)
Age (years)	48.19 ± 22.66
Sex (women, %)	37.04
Antihistamine (%)	
1st generation	46.15
2nd generation	53.85
Hemoglobin (g/dL)	13.63 ± 2.11
White blood cell (× 10^3^/µL)	8.07 ± 2.07
Platelet (× 10^3^/µL)	253.67 ± 95.14
Sodium (mEq/L)	137.04 ± 4.78
Potassium (mEq/L)	4.05 ± 0.42
Blood urea nitrogen (mg/dL)	14.65 ± 5.86
Creatine (mg/dL)	0.98 ± 0.30
Estimated glomerular filtration rate (mL/min)	90.18 ± 19.10
Alanine amino transferase (IU/L)	32.21 ± 23.07
Aspartate aminotransferase (IU/L)	28.50 ± 14.63
C-reactive protein (mg/L)	8.80 ± 19.37
Time lapse^a^ (days)	3.52 ± 3.32

*AIS* antihistamine-induced seizure.

^a^Time lapse indicates the time interval from the first antihistamine doe to the seizure.

### PSD analysis

**Figure 1:**
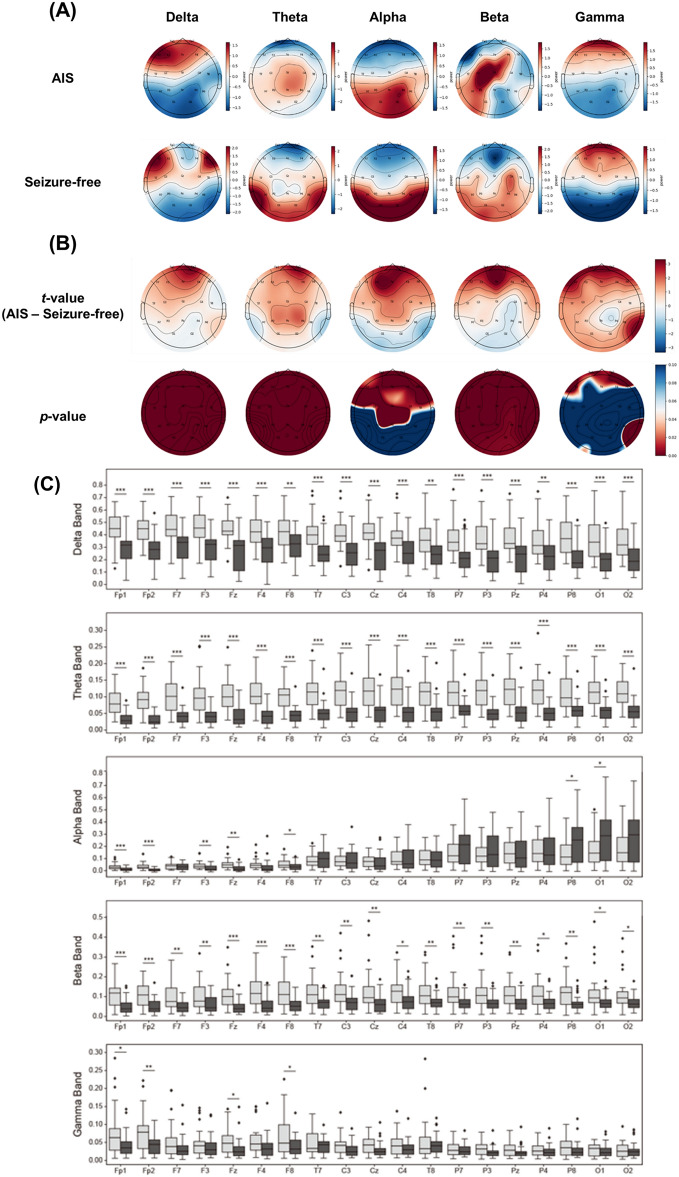
Power spectral density (PSD) analysis. (**A**) Topographical maps of relative PSD in each group (upper panel: antihistamine-induced seizure [AIS] group; lower panel: seizure-free group). (**B**) Topographical maps presenting statistical differences between the AIS and seizure-free groups (upper panel: *t*-value; lower panel: *p*-value). The maps are based on 19 derivations (electrode positions are indicated by channel names). (**C**) Box and whisker plots display the relative PSD values calculated in each frequency band through spectrum analysis. These plots show the differences between the AIS (light gray bars) and seizure-free (dark gray bars) groups across each channel. *P < 0.05, **P < 0.01, ***P < 0.001.

Topographical maps of the relative PSD in each group are presented in Fig. 1A. The maximum PSDs in the AIS group were localized in the frontal areas in the delta and gamma bands, central areas in the theta and beta bands, and parieto-occipital areas in the alpha band (Fig. 1A, upper panel). The maximum PSDs of the seizure-free group were localized in the frontal areas in the delta and gamma bands and parieto-occipital areas in the theta, alpha, and beta bands (Fig. 1A, lower panel). Topographical maps of the differences in relative PSDs between the groups are presented in Fig. 1B. In the topographical maps expressed as *t*-values, the PSD values were higher in the AIS group than in the seizure-free group (Fig. 1B, upper panel). Topographies presenting statistical differences expressed as P-values showed that, compared with the seizure-free group, the AIS group had significantly greater PSD values in global areas in the delta, theta, and beta bands, as well as in the frontocentral areas in the alpha band (Fig. 1B, lower panel). The values of relative PSD in each group are presented in Fig. 1C as box and whisker plots.

### FC and graph theoretical analyses

**Figure 2:**
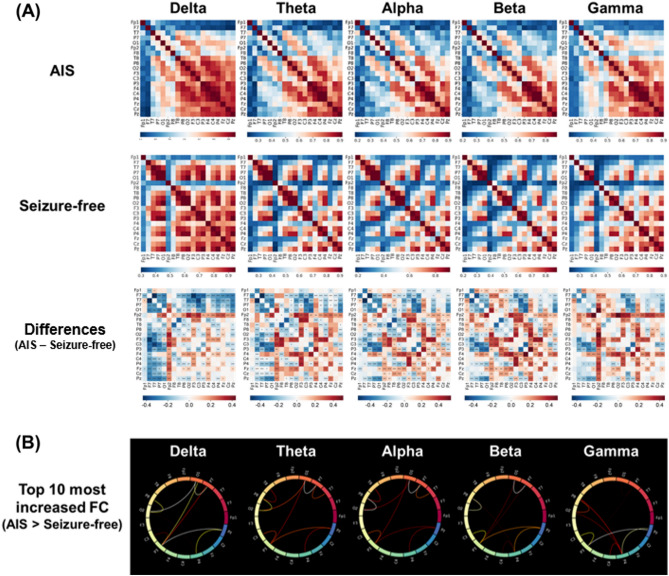
Functional connectivity analysis. (**A**) Functional connectivity adjacent matrices of the coherence between 19 pairs of scalp electroencephalography electrodes in each frequency band. *P < 0.05, **P < 0.01, ***P < 0.001. (**B**) Circular graphs presenting the top 10 increases in functional connectivity in the antihistamine-induced seizure group relative to the seizure-free group.

FCs measured using coherence (Supplementary Table) are presented as adjacent matrices in Fig. 2A. Overall, the FC strength was higher in the AIS group than in the seizure-free group. FCs in the AIS group, compared with those in the seizure-free group, were mainly increased in the parieto-occipital regions (Fig. 2B). Comparisons of the global graph measures between the AIS and seizure-free groups are presented in Table 2. The characteristic path length in the theta band was shorter in the AIS group than in the seizure-free group. In the beta band, the AIS group had higher global efficiency, local efficiency, and clustering coefficients than the seizure-free group (FDR-corrected P < 0.05). The global graph measures did not differ between the groups in the delta, alpha, or gamma bands.

**Table 2:** Comparisons of global graph measures between the antihistamine-induced seizure (AIS) and seizure-free (SF) groups.

Graph measurement	AIS	SF	P	AIS	SF	P	AIS	SF	P	AIS	SF	P	AIS	SF	P
	Delta			Theta			Alpha			Beta			Gamma		
Average degree	14.975	15.695	0.225	13.774	13.232	0.145	12.480	12.793	0.492	13.092	12.814	0.355	14.382	14.428	0.784
Average strength	10.959	12.115	0.154	7.980	7.948	0.703	6.870	6.709	0.691	7.259	6.517	0.067	9.625	9.114	0.331
Radius	6.773	20.828	0.870	3.897	12.579	0.431	4.196	4.337	0.538	4.390	4.607	0.459	7.347	6.659	0.933
Diameter	9.127	22.844	0.842	6.641	15.876	0.385	7.207	7.381	0.493	7.682	8.639	0.085	11.515	11.428	0.838
Characteristic path length	2.358	5.277	0.877	**2.484**	**3.538**	**0.035**	2.761	2.817	0.504	2.783	2.962	0.090	2.935	2.936	0.787
Global efficiency	0.656	0.703	0.197	0.532	0.524	0.420	0.485	0.471	0.321	**0.493**	**0.455**	**0.033**	0.589	0.558	0.250
Local efficiency	2.139	2.440	0.116	1.384	1.410	0.829	1.185	1.107	0.249	**1.226**	**1.046**	**0.022**	1.792	1.589	0.203
Clustering coefficient	0.644	0.698	0.203	0.461	0.473	0.954	0.416	0.398	0.313	**0.433**	**0.383**	**0.012**	0.564	0.532	0.285
Transitivity	1.017	1.103	0.174	0.723	0.757	0.657	0.653	0.628	0.435	0.684	0.611	0.054	0.906	0.863	0.327
Modularity	0.062	0.035	0.088	0.136	0.122	0.572	0.177	0.164	0.495	0.142	0.156	0.398	0.068	0.054	0.409
Assortativity	0.119	0.163	0.444	0.053	0.114	0.078	0.130	0.095	0.248	0.126	0.113	0.774	0.101	0.062	0.210
Small-worldness	0.890	0.887	0.846	0.889	0.946	0.432	0.892	0.891	0.985	0.887	0.902	0.383	1.078	2.529	0.640

Bold font represents statistically significant differences between the groups.

### Performance of machine learning classifiers

**Figure 3:**
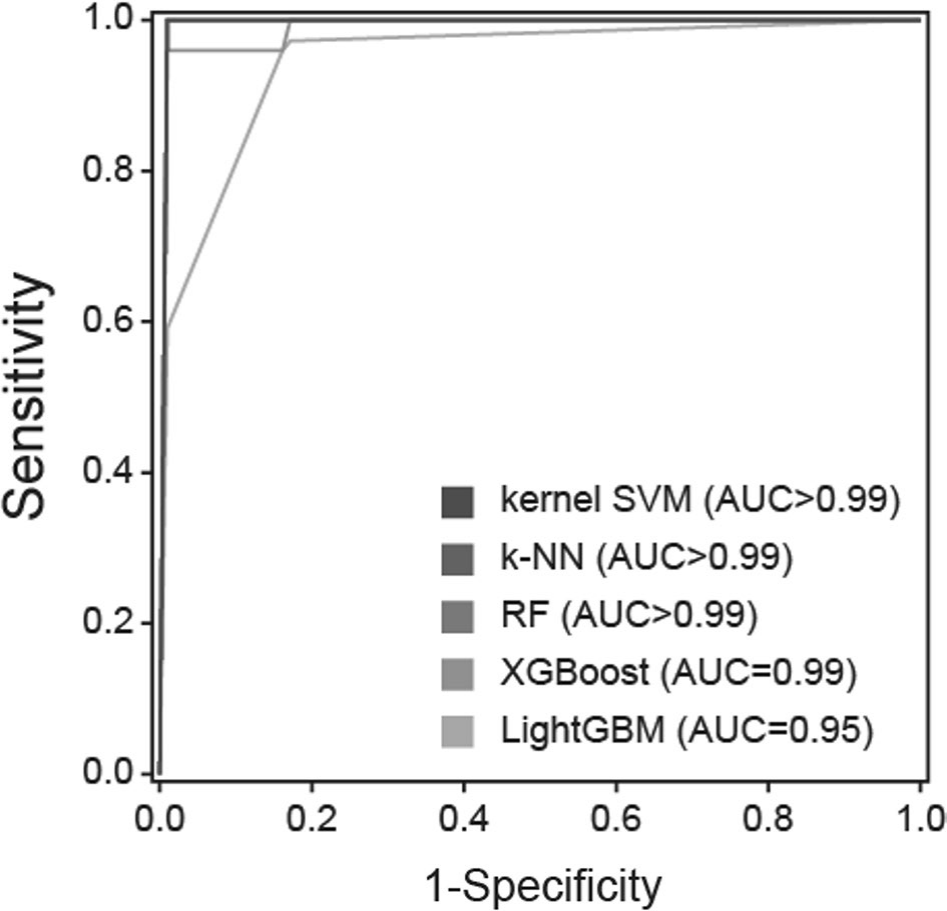
Receiver operating characteristic (ROC) curves. *AUC* area under the curve, *SVM* support vector machine, *k-NN* k-nearest neighbor, *RF* random forest, *XGBoost* extreme gradient boosting, *LightGBM* light gradient boosting machine.

The performance of each machine learning algorithm is presented in Table 3 and Fig. 3. Among the applied algorithms for classification, kernel SVM, k-NN, and RF were found to be the most accurate, with AUC values higher than 0.99, classification accuracies higher than 0.99, and F1 scores higher than 0.99. XGBoost and LightGBM algorithms showed AUC values of 0.99 and 0.95, respectively, as well as classification accuracies of 0.95 and F1 scores of 0.95 each.

**Table 3:** Performance of machine learning models.

Model	AUC	Accuracy	Precision	Recall	F1 score
Kernel support vector machine	> 0.99	> 0.99	> 0.99	> 0.99	> 0.99
k-Nearest neighbor	> 0.99	> 0.99	> 0.99	> 0.99	> 0.99
Random forest	> 0.99	> 0.99	> 0.99	> 0.99	> 0.99
Extreme gradient boosting	0.99	0.95	0.96	0.93	0.95
Light gradient boosting machine	0.95	0.95	0.93	0.97	0.95

*AUC* area under the curve.

## Discussion

We investigated inherent seizure susceptibility in patients with AIS by analyzing the differences in PSD and functional network properties between the AIS and seizure-free groups. The major findings were as follows: (1) compared with the seizure-free group, the AIS group showed higher neural activity during the resting-state in entire areas of the delta, theta, and beta bands, as well as in the frontal areas of the alpha band; (2) the AIS group had higher overall FC strength, and more efficient network properties in the theta and beta bands relative to the seizure-free group; and (3) using the resting-state EEG indices (i.e., PSD, FC, and graph theoretical measurements) as input features, the performance of the machine learning models that differentiated the AIS and seizure-free groups showed a high classification accuracy of more than 99%.

PSD analysis of EEG is a widely used method for quantifying power in a specific frequency band reflecting the process of regional brain activities^27,28^, which has been used for evaluating dynamic changes in neural activity during the initiation and propagation of seizures^29^ and for exploring markers to predict the risk of seizures^30^. According to previous literature^29,30^, increased PSD can represent increases in neural activities, which may be a predisposition to seizure. Therefore, our findings of increased PSD in the entire areas of the delta, theta, and beta bands and in the frontal areas of the alpha band in the AIS group, compared with the seizure-free group, suggest that the AIS group may have inherent seizure susceptibility, even in the resting-state.

However, the pathophysiological mechanisms by which antihistamines induce seizures are not fully understood. An experimental study found that H1-receptor knockout mice experienced longer and more seizures than wild-type mice^7^. In addition, the administration of H1-receptor antagonists increased seizure severity and neuronal damage in the septum, thalamus, hippocampus, and retrosplenial granular cortex of kainic acid-treated wild-type mice^7^. These findings contribute to the knowledge that the central histaminergic neuronal system is a powerful modulator of brain activity, and that its functional disturbance is related to seizure disorders^31^. Moreover, these findings indicate that H1-receptors play a pivotal role in regulating seizure intensity and duration, and that seizure-induced neuronal damage could be localized in selective regions^7,32,33^. Considering that the main EEG generators of the delta and theta frequency bands are the thalamus and hippocampus, respectively^34,35^, PSD increases in entire areas in the delta and theta bands could be associated with seizure susceptibility in selective regions (i.e., the thalamus and hippocampus) in response to antihistamines. In addition, the alpha and beta bands are produced by cortical generators^35^. Thus, increased PSD in the frontal area in the alpha band and in entire regions in the beta band might be related to cortical hyperexcitability, which predisposes to seizures. These findings provide insights into the pathophysiology of AIS, and further studies are required to clarify the underlying mechanisms.

In the functional network analyses, FC strength was found to be increased in the AIS group relative to the seizure-free group. Moreover, compared with the seizure-free group, the AIS group had a decreased characteristic path length in the theta band and increased global efficiency, local efficiency, and clustering coefficient in the beta band. Taken together, these findings suggest that the AIS group has functional network properties that are suitable for efficient transmission of information from the perspective of a large-scale network; therefore, we speculate that these easily synchronizable functional network properties may be responsible for seizure susceptibility in the AIS group. Dynamic changes are known to occur in the network topology during seizure^36^. The ictal period could be characterized by a more synchronized and integrated network configuration of the brain^36–38^. The number of connections gradually declines to preictal levels after the midictal phase, resulting in a less synchronizable and disintegrated network topology after ictal termination^36–38^. Therefore, our finding of a more synchronizable network property in the AIS group may be in accordance with the network configuration of the preictal period or a predisposition to seizures.

We found that kernel Support Vector Machine, k-Nearest Neighbor, Random Forest models based on EEG indices were different between the AIS and seizure-free groups and could classify the groups with a high accuracy of more than 99%. The diagnosis of the side effects of specific drugs, including AISs, can only be confirmed through rechallenge testing in clinical practice^10^; however, the practical application of rechallenge testing is limited owing to possible exposure to physical risk and ethical problems. Considering the above-mentioned limitations of the conventional diagnosis of AIS, our proposed machine learning models based on EEG indices reflecting the seizure susceptibility of AIS could be useful tools for distinguishing patients with AIS from those without, even when using resting-state EEG data without any abnormalities on visual inspection. Moreover, the timely application of our proposed machine learning models may contribute to promoting favorable outcomes, considering the possible adverse effects of the unnecessary use of antiseizure medications in AIS. Our findings of highly accurate performance with more than 99% in most machine learning classifiers suggest that the EEG indices what we used could be generally applicable as important features for classifying the AIS and seizure-free groups.

Our study had several limitations that should be considered. First, the sample size was relatively small. Further studies with larger datasets are required to generalize our results. Second, since our study was cross-sectional, whether our results reflect antihistamine-specific findings or general seizure susceptibility. Future longitudinal studies are required to clarify our results. Finally, because the time interval from seizure to EEG acquisition was different for each patient with AIS, the postictal characteristics might have affected the results of the EEG analyses. However, EEG data were acquired at least 24 h after seizure occurrence, when all patients with AIS showed a normal consciousness level with no postictal neurological abnormalities. Thus, the resting-state EEG data were unlikely to be affected by the influence of postictal characteristics.

We found that patients with AIS had an inherent seizure susceptibility, even during resting-state periods without seizures. Considering that almost everyone has experience with taking antihistamines, our results suggest that individual characteristics (i.e., having seizure susceptibility) may act as a major factor in seizures occurring after taking antihistamines. To the best of our knowledge, this is the first study to investigate inherent seizure susceptibility in patients with acute symptomatic seizure due to antihistamines using resting-state EEG analysis.

## Conclusion

Using resting-state EEG data, we explored markers for differentiating patients with AIS from those without. Our findings show that the AIS group exhibits seizure susceptibility in terms of both regional neural activity and functional network properties. Moreover, based on the EEG indices found to be different between the AIS and seizure-free groups, the machine learning models could differentiate between the groups with a high accuracy of more than 99%. Our results provide insights into the pathophysiological mechanisms underlying AIS and may be useful for the differential diagnosis of new-onset seizures in the clinical setting.

## Supplementary Information

Supplementary Table 1.

## Data Availability

Data are available from the corresponding author upon reasonable request.
