# Supplementary material for: Inherent seizure susceptibility in patients with antihistamine-induced acute symptomatic seizure: a resting-state EEG analysis
Source: Sci Rep. 2023 Jun 5;13:9146. doi: 10.1038/s41598-023-36415-7 (PMC10241146; doi:10.1038/s41598-023-36415-7)
Supplement: Supplementary file 1 — Supplementary Table 1. [file 41598_2023_36415_MOESM1_ESM.pdf]

Supplementary Table

Table. Comparison of the functional connectivity between the antihistamine-induced seizure (AIS) and seizure-free groups. Bold fonts indicate statistically significant results (P < 0.05).

Frequency band	Channels	AIS	Seizure free	*t*-value	P value
delta	Fp1-Fp2	0.40±0.20	0.48±0.19	-1.59	0.12
**delta**	**Fp1-F7**	**0.36±0.22**	**0.55±0.18**	**-3.61**	**< 0.001**
**delta**	**Fp1-F3**	**0.39±0.20**	**0.50±0.19**	**-2.34**	**0.02**
**delta**	**Fp1-Fz**	**0.34±0.20**	**0.45±0.20**	**-2.90**	**0.04**
delta	Fp1-F4	0.34±0.20	0.41±0.18	-1.40	0.17
delta	Fp1-F8	0.37±0.21	0.38±0.19	0.21	0.84
**delta**	**Fp1-T7**	**0.57±0.20**	**0.46±0.20**	**2.90**	**0.04**
delta	Fp1-C3	0.34±0.19	0.44±0.20	-1.78	0.08
delta	Fp1-Cz	0.32±0.19	0.41±0.19	-1.79	0.08
delta	Fp1-C4	0.35±0.21	0.42±0.19	-1.42	0.16
delta	Fp1-T8	0.38±0.20	0.40±0.19	0.37	0.72
delta	Fp1-P7	0.47±0.21	0.42±0.20	0.98	0.33
delta	Fp1-P3	0.35±0.20	0.41±0.20	-1.50	0.30
delta	Fp1-Pz	0.33±0.17	0.40±0.19	-1.41	0.16
delta	Fp1-P4	0.35±0.20	0.40±0.19	-1.20	0.31
delta	Fp1-P8	0.39±0.21	0.38±0.20	0.24	0.81
delta	Fp1-O1	0.43±0.23	0.39±0.20	0.85	0.40
delta	Fp1-O2	0.34±0.23	0.38±0.19	0.60	0.55
delta	Fp2-F7	0.46±0.20	0.42±0.20	0.88	0.38
**delta**	**Fp2-F3**	**0.74±0.21**	**0.45±0.20**	**5.17**	**< 0.001**
**delta**	**Fp2-Fz**	**0.72±0.16**	**0.43±0.21**	**5.91**	**< 0.001**
**delta**	**Fp2-F4**	**0.65±0.21**	**0.48±0.23**	**3.00**	**< 0.001**
**delta**	**Fp2-F8**	**0.70±0.22**	**0.56±0.23**	**2.37**	**0.02**
delta	Fp2-T7	0.40±0.23	0.41±0.20	0.12	0.90
**delta**	**Fp2-C3**	**0.77±0.16**	**0.42±0.20**	**7.28**	**< 0.001**
**delta**	**Fp2-Cz**	**0.60±0.20**	**0.41±0.20**	**3.56**	**< 0.001**
**delta**	**Fp2-C4**	**0.65±0.22**	**0.44±0.21**	**3.65**	**< 0.001**
delta	Fp2-T8	0.59±0.25	0.48±0.22	1.70	0.10
**delta**	**Fp2-P7**	**0.63±0.24**	**0.40±0.20**	**3.84**	**< 0.001**
**delta**	**Fp2-P3**	**0.57±0.23**	**0.41±0.20**	**2.81**	**0.01**
**delta**	**Fp2-Pz**	**0.62±0.16**	**0.40±0.20**	**4.40**	**< 0.001**
**delta**	**Fp2-P4**	**0.69±0.18**	**0.41±0.21**	**5.38**	**< 0.001**
**delta**	**Fp2-P8**	**0.62±0.23**	**0.42±0.21**	**3.47**	**< 0.001**
**delta**	**Fp2-O1**	**0.69±0.24**	**0.39±0.20**	**4.99**	**< 0.001**
**delta**	**Fp2-O2**	**0.76±0.18**	**0.39±0.20**	**7.19**	**< 0.001**

**delta**	**F7-F3**	**0.35±0.20**	**0.78±0.12**	**-10.50**	**< 0.001**
**delta**	**F7-Fz**	**0.32±0.19**	**0.58±0.21**	**-4.91**	**< 0.001**
**delta**	**F7-F4**	**0.33±0.17**	**0.50±0.21**	**-3.50**	**< 0.001**
delta	F7-F8	0.47±0.22	0.48±0.21	0.80	0.93
**delta**	**F7-T7**	**0.36±0.25**	**0.82±0.10**	**-8.87**	**< 0.001**
**delta**	**F7-C3**	**0.38±0.17**	**0.71±0.13**	**-8.26**	**< 0.001**
**delta**	**F7-Cz**	**0.30±0.17**	**0.58±0.20**	**-5.69**	**< 0.001**
**delta**	**F7-C4**	**0.32±0.17**	**0.55±0.21**	**-4.61**	**< 0.001**
**delta**	**F7-T8**	**0.37±0.18**	**0.56±0.20**	**-3.63**	**< 0.001**
**delta**	**F7-P7**	**0.47±0.24**	**0.72±0.14**	**-4.71**	**< 0.001**
**delta**	**F7-P3**	**0.33±0.17**	**0.67±0.15**	**-7.97**	**< 0.001**
**delta**	**F7-Pz**	**0.30±0.17**	**0.60±0.18**	**-6.47**	**< 0.001**
**delta**	**F7-P4**	**0.32±0.17**	**0.57±0.20**	**-4.89**	**< 0.001**
**delta**	**F7-P8**	**0.34±0.19**	**0.57±0.21**	**-4.18**	**< 0.001**
**delta**	**F7-O1**	**0.35±0.20**	**0.64±0.17**	**-5.89**	**< 0.001**
**delta**	**F7-O2**	**0.35±0.17**	**0.59±0.19**	**-5.21**	**< 0.001**
delta	F3-Fz	0.83±0.16	0.79±0.19	0.79	0.44
**delta**	**F3-F4**	**0.83±0.10**	**0.65±0.23**	**3.98**	**< 0.001**
**delta**	**F3-F8**	**0.75±0.16**	**0.52±0.22**	**4.48**	**< 0.001**
**delta**	**F3-T7**	**0.54±0.24**	**0.82±0.12**	**-5.53**	**< 0.001**
delta	F3-C3	0.87±0.10	0.85±0.12	0.69	0.50
delta	F3-Cz	0.76±0.12	0.77±0.19	0.20	0.84
**delta**	**F3-C4**	**0.89±0.08**	**0.71±0.19**	**4.83**	**< 0.001**
delta	F3-T8	0.68±0.19	0.63±0.20	0.92	0.36
**delta**	**F3-P7**	**0.59±0.23**	**0.76±0.13**	**-3.34**	**< 0.001**
delta	F3-P3	0.80±0.15	0.79±0.14	0.20	0.84
delta	F3-Pz	0.81±0.09	0.75±0.16	1.81	0.08
**delta**	**F3-P4**	**0.91±0.07**	**0.71±0.17**	**5.77**	**< 0.001**
**delta**	**F3-P8**	**0.84±0.10**	**0.66±0.19**	**4.41**	**< 0.001**
**delta**	**F3-O1**	**0.80±0.12**	**0.71±0.15**	**2.21**	**0.03**
**delta**	**F3-O2**	**0.85±0.12**	**0.68±0.16**	**4.37**	**< 0.001**
delta	Fz-F4	0.82±0.13	0.77±0.19	1.17	0.25
delta	Fz-F8	0.65±0.17	0.57±0.22	1.71	0.09
**delta**	**Fz-T7**	**0.48±0.23**	**0.69±0.18**	**-3.89**	**< 0.001**
delta	Fz-C3	0.84±0.15	0.79±0.17	1.50	0.30
delta	Fz-Cz	0.86±0.08	0.86±0.13	0.10	1.00
delta	Fz-C4	0.82±0.15	0.79±0.17	0.86	0.39
delta	Fz-T8	0.69±0.18	0.68±0.18	0.70	0.95
**delta**	**Fz-P7**	**0.56±0.21**	**0.69±0.18**	**-2.58**	**0.01**
delta	Fz-P3	0.79±0.14	0.76±0.16	0.87	0.39

**delta**	**Fz-Pz**	**0.86±0.11**	**0.77±0.17**	**2.45**	**0.02**
**delta**	**Fz-P4**	**0.87±0.16**	**0.75±0.17**	**2.58**	**0.01**
**delta**	**Fz-P8**	**0.77±0.13**	**0.69±0.18**	**2.20**	**0.05**
delta	Fz-O1	0.72±0.14	0.69±0.17	0.61	0.54
**delta**	**Fz-O2**	**0.80±0.12**	**0.69±0.17**	**2.75**	**0.01**
**delta**	**F4-F8**	**0.56±0.21**	**0.74±0.16**	**-3.70**	**< 0.001**
delta	F4-T7	0.60±0.18	0.62±0.22	0.47	0.64
delta	F4-C3	0.78±0.14	0.70±0.20	1.90	0.06
**delta**	**F4-Cz**	**0.89±0.06**	**0.76±0.21**	**3.32**	**< 0.001**
**delta**	**F4-C4**	**0.92±0.08**	**0.82±0.15**	**3.22**	**< 0.001**
delta	F4-T8	0.84±0.09	0.79±0.14	1.58	0.12
delta	F4-P7	0.70±0.18	0.65±0.20	1.70	0.29
**delta**	**F4-P3**	**0.92±0.05**	**0.70±0.20**	**5.99**	**< 0.001**
**delta**	**F4-Pz**	**0.87±0.07**	**0.74±0.18**	**3.84**	**< 0.001**
**delta**	**F4-P4**	**0.91±0.06**	**0.76±0.17**	**4.58**	**< 0.001**
**delta**	**F4-P8**	**0.90±0.08**	**0.73±0.18**	**4.73**	**< 0.001**
delta	F4-O1	0.73±0.13	0.66±0.20	1.58	0.12
**delta**	**F4-O2**	**0.89±0.09**	**0.69±0.20**	**5.60**	**< 0.001**
delta	F8-T7	0.50±0.24	0.56±0.22	-1.50	0.30
**delta**	**F8-C3**	**0.79±0.13**	**0.55±0.23**	**4.80**	**< 0.001**
delta	F8-Cz	0.54±0.20	0.57±0.24	0.51	0.61
**delta**	**F8-C4**	**0.61±0.21**	**0.71±0.16**	**-2.40**	**0.05**
**delta**	**F8-T8**	**0.48±0.22**	**0.82±0.12**	**-7.90**	**< 0.001**
**delta**	**F8-P7**	**0.41±0.21**	**0.58±0.22**	**-2.89**	**0.01**
delta	F8-P3	0.56±0.25	0.58±0.22	0.18	0.86
delta	F8-Pz	0.61±0.17	0.59±0.21	0.38	0.71
delta	F8-P4	0.66±0.17	0.65±0.20	0.20	0.84
**delta**	**F8-P8**	**0.58±0.24**	**0.69±0.18**	**-2.80**	**0.04**
delta	F8-O1	0.60±0.22	0.59±0.22	0.20	0.84
delta	F8-O2	0.58±0.19	0.62±0.21	0.68	0.50
**delta**	**T7-C3**	**0.46±0.22**	**0.88±0.08**	**-9.39**	**< 0.001**
**delta**	**T7-Cz**	**0.56±0.17**	**0.75±0.18**	**-4.26**	**< 0.001**
**delta**	**T7-C4**	**0.56±0.20**	**0.74±0.18**	**-3.56**	**< 0.001**
delta	T7-T8	0.71±0.24	0.73±0.20	0.41	0.68
**delta**	**T7-P7**	**0.68±0.24**	**0.92±0.05**	**-5.90**	**< 0.001**
**delta**	**T7-P3**	**0.68±0.15**	**0.88±0.07**	**-6.63**	**< 0.001**
**delta**	**T7-Pz**	**0.52±0.19**	**0.81±0.11**	**-7.50**	**< 0.001**
**delta**	**T7-P4**	**0.52±0.22**	**0.78±0.14**	**-5.40**	**< 0.001**
**delta**	**T7-P8**	**0.68±0.17**	**0.79±0.14**	**-2.52**	**0.01**
**delta**	**T7-O1**	**0.60±0.23**	**0.86±0.08**	**-5.63**	**< 0.001**

**delta**	**T7-O2**	**0.51±0.22**	**0.81±0.11**	**-6.49**	**< 0.001**
**delta**	**C3-Cz**	**0.75±0.11**	**0.88±0.16**	**-3.51**	**< 0.001**
delta	C3-C4	0.81±0.11	0.83±0.16	0.51	0.61
delta	C3-T8	0.70±0.22	0.74±0.17	0.83	0.41
**delta**	**C3-P7**	**0.56±0.22**	**0.88±0.07**	**-7.45**	**< 0.001**
**delta**	**C3-P3**	**0.74±0.16**	**0.93±0.06**	**-6.11**	**< 0.001**
**delta**	**C3-Pz**	**0.80±0.08**	**0.90±0.07**	**-5.30**	**< 0.001**
delta	C3-P4	0.88±0.08	0.86±0.10	0.83	0.41
**delta**	**C3-P8**	**0.71±0.15**	**0.80±0.12**	**-2.39**	**0.02**
**delta**	**C3-O1**	**0.66±0.17**	**0.86±0.08**	**-5.51**	**< 0.001**
delta	C3-O2	0.78±0.13	0.83±0.10	-1.82	0.08
delta	Cz-C4	0.84±0.10	0.87±0.18	0.77	0.45
delta	Cz-T8	0.77±0.11	0.74±0.19	0.60	0.55
**delta**	**Cz-P7**	**0.59±0.17**	**0.78±0.17**	**-4.33**	**< 0.001**
delta	Cz-P3	0.90±0.06	0.87±0.16	0.88	0.38
delta	Cz-Pz	0.91±0.06	0.90±0.16	0.14	0.89
delta	Cz-P4	0.84±0.09	0.86±0.16	0.57	0.57
delta	Cz-P8	0.82±0.08	0.79±0.17	0.85	0.40
**delta**	**Cz-O1**	**0.67±0.14**	**0.80±0.16**	**-3.30**	**< 0.001**
delta	Cz-O2	0.78±0.13	0.80±0.16	0.54	0.59
**delta**	**C4-T8**	**0.75±0.14**	**0.89±0.06**	**-4.58**	**< 0.001**
**delta**	**C4-P7**	**0.64±0.21**	**0.78±0.16**	**-2.80**	**0.01**
delta	C4-P3	0.87±0.09	0.85±0.14	0.75	0.46
delta	C4-Pz	0.87±0.07	0.90±0.11	-1.25	0.22
delta	C4-P4	0.94±0.06	0.92±0.09	0.97	0.34
delta	C4-P8	0.87±0.10	0.88±0.08	0.42	0.68
delta	C4-O1	0.74±0.15	0.81±0.14	-1.89	0.06
delta	C4-O2	0.87±0.14	0.85±0.12	0.52	0.61
delta	T8-P7	0.79±0.13	0.78±0.15	0.36	0.72
delta	T8-P3	0.85±0.12	0.78±0.14	1.94	0.06
**delta**	**T8-Pz**	**0.73±0.14**	**0.80±0.12**	**-2.15**	**0.04**
**delta**	**T8-P4**	**0.75±0.15**	**0.87±0.09**	**-3.55**	**< 0.001**
**delta**	**T8-P8**	**0.80±0.14**	**0.91±0.06**	**-3.93**	**< 0.001**
**delta**	**T8-O1**	**0.62±0.20**	**0.80±0.12**	**-3.83**	**< 0.001**
**delta**	**T8-O2**	**0.75±0.17**	**0.84±0.10**	**-2.54**	**0.01**
**delta**	**P7-P3**	**0.67±0.21**	**0.93±0.04**	**-6.48**	**< 0.001**
**delta**	**P7-Pz**	**0.56±0.17**	**0.87±0.07**	**-8.62**	**< 0.001**
**delta**	**P7-P4**	**0.62±0.20**	**0.85±0.09**	**-5.52**	**< 0.001**
**delta**	**P7-P8**	**0.73±0.25**	**0.86±0.09**	**-2.62**	**0.01**
**delta**	**P7-O1**	**0.66±0.28**	**0.95±0.03**	**-5.34**	**< 0.001**

**delta**	**P7-O2**	**0.71±0.19**	**0.90±0.06**	**-4.78**	**< 0.001**
**delta**	**P3-Pz**	**0.86±0.10**	**0.95±0.03**	**-4.74**	**< 0.001**
**delta**	**P3-P4**	**0.85±0.09**	**0.91±0.07**	**-2.93**	**0.01**
delta	P3-P8	0.89±0.07	0.87±0.08	1.22	0.23
**delta**	**P3-O1**	**0.70±0.16**	**0.94±0.03**	**-7.89**	**< 0.001**
**delta**	**P3-O2**	**0.79±0.14**	**0.91±0.04**	**-4.27**	**< 0.001**
**delta**	**Pz-P4**	**0.89±0.06**	**0.94±0.05**	**-3.84**	**< 0.001**
**delta**	**Pz-P8**	**0.79±0.09**	**0.88±0.08**	**-3.84**	**< 0.001**
**delta**	**Pz-O1**	**0.66±0.13**	**0.90±0.06**	**-8.99**	**< 0.001**
**delta**	**Pz-O2**	**0.78±0.12**	**0.91±0.05**	**-5.13**	**< 0.001**
**delta**	**P4-P8**	**0.83±0.10**	**0.93±0.06**	**-4.44**	**< 0.001**
**delta**	**P4-O1**	**0.73±0.15**	**0.89±0.08**	**-5.17**	**< 0.001**
**delta**	**P4-O2**	**0.86±0.10**	**0.93±0.06**	**-2.96**	**< 0.001**
**delta**	**P8-O1**	**0.82±0.15**	**0.89±0.07**	**-2.54**	**0.02**
**delta**	**P8-O2**	**0.87±0.12**	**0.94±0.04**	**-3.19**	**< 0.001**
**delta**	**O1-O2**	**0.80±0.15**	**0.95±0.03**	**-4.94**	**< 0.001**
theta	Fp1-Fp2	0.28±0.16	0.28±0.17	0.20	0.98
**theta**	**Fp1-F7**	**0.29±0.18**	**0.60±0.17**	**-6.57**	**< 0.001**
**theta**	**Fp1-F3**	**0.27±0.12**	**0.39±0.16**	**-3.32**	**< 0.001**
**theta**	**Fp1-Fz**	**0.24±0.13**	**0.32±0.16**	**-2.12**	**0.04**
theta	Fp1-F4	0.28±0.13	0.28±0.14	0.10	0.99
theta	Fp1-F8	0.28±0.13	0.24±0.13	1.27	0.21
**theta**	**Fp1-T7**	**0.62±0.16**	**0.44±0.16**	**4.34**	**< 0.001**
**theta**	**Fp1-C3**	**0.23±0.10**	**0.33±0.17**	**-2.65**	**0.01**
theta	Fp1-Cz	0.28±0.12	0.25±0.16	0.75	0.46
theta	Fp1-C4	0.26±0.13	0.25±0.16	0.48	0.63
**theta**	**Fp1-T8**	**0.40±0.17**	**0.24±0.14**	**3.94**	**< 0.001**
theta	Fp1-P7	0.42±0.23	0.34±0.15	1.52	0.13
theta	Fp1-P3	0.34±0.12	0.29±0.14	1.24	0.22
theta	Fp1-Pz	0.24±0.10	0.24±0.15	0.60	0.95
theta	Fp1-P4	0.24±0.10	0.24±0.14	0.40	0.97
**theta**	**Fp1-P8**	**0.35±0.18**	**0.24±0.13**	**2.62**	**0.01**
**theta**	**Fp1-O1**	**0.39±0.20**	**0.29±0.13**	**2.22**	**0.03**
theta	Fp1-O2	0.25±0.17	0.25±0.13	0.30	0.97
**theta**	**Fp2-F7**	**0.49±0.19**	**0.26±0.14**	**5.26**	**< 0.001**
**theta**	**Fp2-F3**	**0.65±0.26**	**0.30±0.17**	**5.94**	**< 0.001**
**theta**	**Fp2-Fz**	**0.60±0.15**	**0.30±0.16**	**7.39**	**< 0.001**
**theta**	**Fp2-F4**	**0.50±0.13**	**0.36±0.16**	**3.53**	**< 0.001**
theta	Fp2-F8	0.59±0.25	0.59±0.17	0.11	0.91
theta	Fp2-T7	0.24±0.14	0.24±0.14	0.80	0.94

**theta**	**Fp2-C3**	**0.61±0.16**	**0.23±0.15**	**9.10**	**< 0.001**
**theta**	**Fp2-Cz**	**0.45±0.17**	**0.24±0.15**	**4.98**	**< 0.001**
**theta**	**Fp2-C4**	**0.50±0.17**	**0.30±0.13**	**4.94**	**< 0.001**
theta	Fp2-T8	0.38±0.17	0.43±0.15	-1.18	0.24
**theta**	**Fp2-P7**	**0.47±0.22**	**0.24±0.14**	**4.63**	**< 0.001**
**theta**	**Fp2-P3**	**0.38±0.14**	**0.22±0.14**	**4.20**	**< 0.001**
**theta**	**Fp2-Pz**	**0.46±0.13**	**0.22±0.14**	**6.83**	**< 0.001**
**theta**	**Fp2-P4**	**0.54±0.18**	**0.27±0.13**	**6.41**	**< 0.001**
**theta**	**Fp2-P8**	**0.50±0.18**	**0.33±0.14**	**4.12**	**< 0.001**
**theta**	**Fp2-O1**	**0.62±0.22**	**0.24±0.13**	**7.69**	**< 0.001**
**theta**	**Fp2-O2**	**0.69±0.14**	**0.27±0.13**	**11.88**	**< 0.001**
**theta**	**F7-F3**	**0.32±0.18**	**0.63±0.13**	**-7.35**	**< 0.001**
theta	F7-Fz	0.32±0.13	0.30±0.16	0.42	0.68
theta	F7-F4	0.24±0.11	0.28±0.15	-1.20	0.24
**theta**	**F7-F8**	**0.53±0.23**	**0.30±0.13**	**4.53**	**< 0.001**
**theta**	**F7-T7**	**0.27±0.16**	**0.80±0.07**	**-15.87**	**< 0.001**
**theta**	**F7-C3**	**0.41±0.16**	**0.59±0.12**	**-4.73**	**< 0.001**
**theta**	**F7-Cz**	**0.24±0.11**	**0.32±0.18**	**-2.29**	**0.03**
**theta**	**F7-C4**	**0.24±0.12**	**0.33±0.16**	**-2.28**	**0.03**
**theta**	**F7-T8**	**0.28±0.13**	**0.37±0.16**	**-2.49**	**0.02**
**theta**	**F7-P7**	**0.41±0.21**	**0.64±0.10**	**-5.28**	**< 0.001**
**theta**	**F7-P3**	**0.22±0.10**	**0.54±0.11**	**-11.53**	**< 0.001**
**theta**	**F7-Pz**	**0.25±0.10**	**0.41±0.14**	**-4.84**	**< 0.001**
**theta**	**F7-P4**	**0.27±0.13**	**0.38±0.15**	**-2.91**	**0.01**
**theta**	**F7-P8**	**0.25±0.15**	**0.41±0.15**	**-4.16**	**< 0.001**
**theta**	**F7-O1**	**0.32±0.19**	**0.52±0.10**	**-5.20**	**< 0.001**
**theta**	**F7-O2**	**0.29±0.16**	**0.45±0.13**	**-4.25**	**< 0.001**
theta	F3-Fz	0.69±0.22	0.64±0.21	0.73	0.47
**theta**	**F3-F4**	**0.72±0.11**	**0.40±0.21**	**7.21**	**< 0.001**
**theta**	**F3-F8**	**0.71±0.14**	**0.28±0.17**	**10.54**	**< 0.001**
**theta**	**F3-T7**	**0.40±0.19**	**0.60±0.13**	**-4.62**	**< 0.001**
theta	F3-C3	0.78±0.12	0.76±0.10	0.84	0.41
theta	F3-Cz	0.58±0.13	0.63±0.19	-1.80	0.28
**theta**	**F3-C4**	**0.85±0.07**	**0.46±0.18**	**10.97**	**< 0.001**
**theta**	**F3-T8**	**0.53±0.16**	**0.32±0.17**	**4.93**	**< 0.001**
theta	F3-P7	0.46±0.19	0.49±0.14	0.71	0.48
theta	F3-P3	0.66±0.15	0.59±0.13	1.86	0.07
**theta**	**F3-Pz**	**0.67±0.12**	**0.55±0.14**	**3.36**	**< 0.001**
**theta**	**F3-P4**	**0.85±0.10**	**0.44±0.15**	**12.38**	**< 0.001**
**theta**	**F3-P8**	**0.70±0.12**	**0.34±0.16**	**9.61**	**< 0.001**

**theta**	**F3-O1**	**0.61±0.17**	**0.43±0.14**	**4.19**	**< 0.001**
**theta**	**F3-O2**	**0.73±0.20**	**0.38±0.15**	**7.37**	**< 0.001**
theta	Fz-F4	0.69±0.15	0.62±0.19	1.57	0.12
**theta**	**Fz-F8**	**0.54±0.18**	**0.30±0.17**	**5.23**	**< 0.001**
theta	Fz-T7	0.33±0.15	0.34±0.18	0.16	0.87
**theta**	**Fz-C3**	**0.74±0.25**	**0.57±0.19**	**2.84**	**0.01**
theta	Fz-Cz	0.79±0.09	0.75±0.14	1.29	0.20
**theta**	**Fz-C4**	**0.70±0.22**	**0.55±0.19**	**2.59**	**0.01**
**theta**	**Fz-T8**	**0.51±0.18**	**0.33±0.18**	**3.82**	**< 0.001**
theta	Fz-P7	0.34±0.13	0.34±0.17	0.60	0.96
**theta**	**Fz-P3**	**0.65±0.14**	**0.45±0.19**	**4.73**	**< 0.001**
**theta**	**Fz-Pz**	**0.81±0.15**	**0.52±0.19**	**6.48**	**< 0.001**
**theta**	**Fz-P4**	**0.77±0.26**	**0.45±0.19**	**5.26**	**< 0.001**
**theta**	**Fz-P8**	**0.60±0.13**	**0.33±0.18**	**6.72**	**< 0.001**
**theta**	**Fz-O1**	**0.49±0.20**	**0.34±0.17**	**3.18**	**< 0.001**
**theta**	**Fz-O2**	**0.62±0.12**	**0.33±0.17**	**7.50**	**< 0.001**
**theta**	**F4-F8**	**0.44±0.16**	**0.58±0.14**	**-3.49**	**< 0.001**
**theta**	**F4-T7**	**0.51±0.15**	**0.31±0.19**	**4.42**	**< 0.001**
**theta**	**F4-C3**	**0.69±0.14**	**0.47±0.20**	**4.82**	**< 0.001**
**theta**	**F4-Cz**	**0.81±0.10**	**0.62±0.17**	**5.26**	**< 0.001**
**theta**	**F4-C4**	**0.88±0.12**	**0.72±0.12**	**4.86**	**< 0.001**
**theta**	**F4-T8**	**0.78±0.09**	**0.56±0.15**	**7.17**	**< 0.001**
**theta**	**F4-P7**	**0.58±0.12**	**0.32±0.19**	**6.26**	**< 0.001**
**theta**	**F4-P3**	**0.88±0.08**	**0.44±0.19**	**11.46**	**< 0.001**
**theta**	**F4-Pz**	**0.81±0.07**	**0.55±0.17**	**7.76**	**< 0.001**
**theta**	**F4-P4**	**0.86±0.06**	**0.56±0.16**	**9.80**	**< 0.001**
**theta**	**F4-P8**	**0.82±0.12**	**0.47±0.18**	**8.84**	**< 0.001**
**theta**	**F4-O1**	**0.52±0.14**	**0.35±0.19**	**3.86**	**< 0.001**
**theta**	**F4-O2**	**0.80±0.09**	**0.41±0.19**	**10.30**	**< 0.001**
theta	F8-T7	0.39±0.26	0.38±0.16	0.16	0.87
**theta**	**F8-C3**	**0.74±0.16**	**0.33±0.17**	**9.50**	**< 0.001**
theta	F8-Cz	0.42±0.14	0.33±0.18	1.99	0.05
theta	F8-C4	0.53±0.17	0.59±0.13	-1.43	0.16
**theta**	**F8-T8**	**0.38±0.18**	**0.80±0.05**	**-11.32**	**< 0.001**
**theta**	**F8-P7**	**0.29±0.16**	**0.40±0.13**	**-2.89**	**0.01**
theta	F8-P3	0.47±0.22	0.38±0.14	1.80	0.08
**theta**	**F8-Pz**	**0.51±0.14**	**0.41±0.15**	**2.56**	**0.01**
theta	F8-P4	0.57±0.12	0.54±0.12	1.70	0.29
**theta**	**F8-P8**	**0.44±0.22**	**0.62±0.09**	**-4.15**	**< 0.001**
theta	F8-O1	0.41±0.19	0.42±0.11	0.25	0.80

**theta**	**F8-O2**	**0.41±0.16**	**0.50±0.09**	**-2.60**	**0.01**
**theta**	**T7-C3**	**0.33±0.16**	**0.77±0.09**	**-12.59**	**< 0.001**
theta	T7-Cz	0.44±0.14	0.47±0.17	0.67	0.50
theta	T7-C4	0.46±0.12	0.49±0.16	0.83	0.41
**theta**	**T7-T8**	**0.68±0.25**	**0.55±0.16**	**2.27**	**0.03**
**theta**	**T7-P7**	**0.60±0.25**	**0.89±0.03**	**-5.85**	**< 0.001**
**theta**	**T7-P3**	**0.60±0.11**	**0.80±0.06**	**-7.93**	**< 0.001**
**theta**	**T7-Pz**	**0.39±0.12**	**0.63±0.11**	**-7.64**	**< 0.001**
**theta**	**T7-P4**	**0.40±0.13**	**0.59±0.15**	**-5.60**	**< 0.001**
theta	T7-P8	0.61±0.14	0.62±0.12	0.31	0.76
**theta**	**T7-O1**	**0.43±0.19**	**0.78±0.06**	**-9.11**	**< 0.001**
**theta**	**T7-O2**	**0.37±0.18**	**0.68±0.09**	**-7.93**	**< 0.001**
**theta**	**C3-Cz**	**0.59±0.13**	**0.78±0.16**	**-4.83**	**< 0.001**
theta	C3-C4	0.71±0.11	0.68±0.15	0.89	0.38
**theta**	**C3-T8**	**0.60±0.21**	**0.50±0.16**	**2.11**	**0.04**
**theta**	**C3-P7**	**0.41±0.19**	**0.74±0.10**	**-8.43**	**< 0.001**
**theta**	**C3-P3**	**0.60±0.12**	**0.89±0.04**	**-11.77**	**< 0.001**
**theta**	**C3-Pz**	**0.71±0.11**	**0.84±0.07**	**-5.18**	**< 0.001**
**theta**	**C3-P4**	**0.83±0.08**	**0.70±0.12**	**4.85**	**< 0.001**
theta	C3-P8	0.51±0.15	0.57±0.14	-1.48	0.15
**theta**	**C3-O1**	**0.39±0.18**	**0.71±0.10**	**-8.10**	**< 0.001**
theta	C3-O2	0.60±0.14	0.64±0.11	-1.17	0.25
theta	Cz-C4	0.73±0.17	0.77±0.16	-1.40	0.30
**theta**	**Cz-T8**	**0.65±0.17**	**0.47±0.18**	**4.16**	**< 0.001**
**theta**	**Cz-P7**	**0.40±0.13**	**0.49±0.17**	**-2.35**	**0.02**
**theta**	**Cz-P3**	**0.84±0.11**	**0.70±0.15**	**3.94**	**< 0.001**
theta	Cz-Pz	0.87±0.06	0.82±0.14	1.80	0.08
theta	Cz-P4	0.72±0.16	0.70±0.15	0.39	0.70
**theta**	**Cz-P8**	**0.70±0.11**	**0.50±0.17**	**5.35**	**< 0.001**
theta	Cz-O1	0.45±0.19	0.53±0.17	-1.73	0.09
**theta**	**Cz-O2**	**0.63±0.14**	**0.54±0.15**	**2.42**	**0.02**
**theta**	**C4-T8**	**0.66±0.11**	**0.75±0.11**	**-2.93**	**< 0.001**
theta	C4-P7	0.52±0.14	0.54±0.16	0.50	0.62
**theta**	**C4-P3**	**0.81±0.07**	**0.69±0.15**	**4.60**	**< 0.001**
theta	C4-Pz	0.78±0.11	0.82±0.11	-1.19	0.24
**theta**	**C4-P4**	**0.91±0.07**	**0.86±0.09**	**2.58**	**0.01**
theta	C4-P8	0.77±0.13	0.72±0.12	1.43	0.16
theta	C4-O1	0.53±0.16	0.59±0.16	-1.39	0.17
theta	C4-O2	0.76±0.19	0.68±0.14	1.77	0.08
**theta**	**T8-P7**	**0.70±0.12**	**0.61±0.12**	**2.78**	**0.01**

**theta**	**T8-P3**	**0.79±0.18**	**0.59±0.11**	**5.00**	**< 0.001**
theta	T8-Pz	0.61±0.10	0.62±0.12	0.20	0.84
**theta**	**T8-P4**	**0.65±0.12**	**0.77±0.10**	**-4.10**	**< 0.001**
**theta**	**T8-P8**	**0.69±0.19**	**0.88±0.04**	**-4.99**	**< 0.001**
**theta**	**T8-O1**	**0.39±0.19**	**0.64±0.10**	**-6.17**	**< 0.001**
**theta**	**T8-O2**	**0.60±0.14**	**0.75±0.07**	**-5.60**	**< 0.001**
**theta**	**P7-P3**	**0.51±0.16**	**0.86±0.05**	**-10.76**	**< 0.001**
**theta**	**P7-Pz**	**0.37±0.09**	**0.69±0.12**	**-11.36**	**< 0.001**
**theta**	**P7-P4**	**0.48±0.12**	**0.67±0.14**	**-5.45**	**< 0.001**
theta	P7-P8	0.66±0.27	0.72±0.10	-1.12	0.27
**theta**	**P7-O1**	**0.59±0.28**	**0.92±0.03**	**-6.70**	**< 0.001**
**theta**	**P7-O2**	**0.63±0.16**	**0.79±0.07**	**-4.94**	**< 0.001**
**theta**	**P3-Pz**	**0.80±0.07**	**0.89±0.05**	**-5.51**	**< 0.001**
theta	P3-P4	0.74±0.10	0.79±0.12	-1.83	0.07
**theta**	**P3-P8**	**0.80±0.08**	**0.70±0.09**	**4.50**	**< 0.001**
**theta**	**P3-O1**	**0.46±0.15**	**0.88±0.05**	**-13.70**	**< 0.001**
**theta**	**P3-O2**	**0.63±0.12**	**0.80±0.06**	**-6.72**	**< 0.001**
**theta**	**Pz-P4**	**0.81±0.12**	**0.88±0.09**	**-2.35**	**0.02**
theta	Pz-P8	0.64±0.12	0.70±0.12	-1.81	0.08
**theta**	**Pz-O1**	**0.41±0.15**	**0.76±0.11**	**-9.86**	**< 0.001**
**theta**	**Pz-O2**	**0.63±0.15**	**0.78±0.09**	**-4.63**	**< 0.001**
**theta**	**P4-P8**	**0.68±0.14**	**0.85±0.12**	**-4.78**	**< 0.001**
**theta**	**P4-O1**	**0.49±0.17**	**0.75±0.14**	**-6.35**	**< 0.001**
**theta**	**P4-O2**	**0.73±0.14**	**0.85±0.13**	**-3.23**	**< 0.001**
theta	P8-O1	0.71±0.15	0.78±0.08	-1.95	0.06
**theta**	**P8-O2**	**0.80±0.13**	**0.91±0.04**	**-3.91**	**< 0.001**
**theta**	**O1-O2**	**0.71±0.18**	**0.89±0.05**	**-5.70**	**< 0.001**
alpha	Fp1-Fp2	0.27±0.14	0.27±0.15	0.20	0.99
**alpha**	**Fp1-F7**	**0.29±0.14**	**0.67±0.13**	**-10.37**	**< 0.001**
**alpha**	**Fp1-F3**	**0.25±0.10**	**0.40±0.17**	**-4.40**	**< 0.001**
**alpha**	**Fp1-Fz**	**0.23±0.10**	**0.36±0.14**	**-4.10**	**< 0.001**
**alpha**	**Fp1-F4**	**0.24±0.10**	**0.31±0.13**	**-2.11**	**0.04**
alpha	Fp1-F8	0.27±0.13	0.24±0.11	0.95	0.35
**alpha**	**Fp1-T7**	**0.62±0.15**	**0.49±0.15**	**3.11**	**< 0.001**
**alpha**	**Fp1-C3**	**0.21±0.07**	**0.35±0.15**	**-4.57**	**< 0.001**
alpha	Fp1-Cz	0.25±0.10	0.29±0.14	-1.70	0.29
alpha	Fp1-C4	0.23±0.09	0.25±0.14	0.61	0.55
**alpha**	**Fp1-T8**	**0.36±0.16**	**0.26±0.12**	**2.79**	**0.01**
alpha	Fp1-P7	0.40±0.21	0.39±0.13	0.26	0.79
alpha	Fp1-P3	0.29±0.10	0.32±0.13	0.72	0.48

alpha	Fp1-Pz	0.21±0.08	0.25±0.13	-1.20	0.24
alpha	Fp1-P4	0.21±0.06	0.25±0.12	-1.47	0.15
alpha	Fp1-P8	0.34±0.16	0.27±0.12	1.72	0.09
alpha	Fp1-O1	0.41±0.20	0.32±0.12	1.98	0.05
alpha	Fp1-O2	0.24±0.14	0.28±0.11	-1.19	0.24
**alpha**	**Fp2-F7**	**0.48±0.19**	**0.24±0.11**	**5.63**	**< 0.001**
**alpha**	**Fp2-F3**	**0.60±0.27**	**0.30±0.15**	**5.16**	**< 0.001**
**alpha**	**Fp2-Fz**	**0.54±0.17**	**0.33±0.15**	**5.50**	**< 0.001**
alpha	Fp2-F4	0.41±0.13	0.34±0.18	1.67	0.10
alpha	Fp2-F8	0.55±0.25	0.64±0.15	-1.68	0.10
alpha	Fp2-T7	0.22±0.11	0.23±0.11	0.26	0.79
**alpha**	**Fp2-C3**	**0.55±0.17**	**0.22±0.12**	**8.31**	**< 0.001**
**alpha**	**Fp2-Cz**	**0.37±0.18**	**0.25±0.12**	**2.86**	**0.01**
**alpha**	**Fp2-C4**	**0.42±0.19**	**0.27±0.12**	**3.53**	**< 0.001**
**alpha**	**Fp2-T8**	**0.30±0.16**	**0.45±0.16**	**-3.48**	**< 0.001**
**alpha**	**Fp2-P7**	**0.43±0.22**	**0.25±0.12**	**3.80**	**< 0.001**
alpha	Fp2-P3	0.28±0.14	0.22±0.09	1.95	0.06
**alpha**	**Fp2-Pz**	**0.37±0.15**	**0.21±0.10**	**4.43**	**< 0.001**
**alpha**	**Fp2-P4**	**0.48±0.18**	**0.26±0.12**	**5.21**	**< 0.001**
alpha	Fp2-P8	0.41±0.19	0.34±0.13	1.66	0.10
**alpha**	**Fp2-O1**	**0.61±0.23**	**0.25±0.12**	**7.46**	**< 0.001**
**alpha**	**Fp2-O2**	**0.64±0.14**	**0.28±0.11**	**10.76**	**< 0.001**
**alpha**	**F7-F3**	**0.29±0.15**	**0.58±0.12**	**-8.22**	**< 0.001**
alpha	F7-Fz	0.31±0.13	0.28±0.10	1.40	0.30
alpha	F7-F4	0.20±0.08	0.23±0.10	0.97	0.33
**alpha**	**F7-F8**	**0.53±0.24**	**0.25±0.11**	**5.65**	**< 0.001**
**alpha**	**F7-T7**	**0.21±0.12**	**0.79±0.07**	**-21.16**	**< 0.001**
**alpha**	**F7-C3**	**0.40±0.14**	**0.55±0.10**	**-4.60**	**< 0.001**
alpha	F7-Cz	0.22±0.10	0.26±0.10	-1.82	0.07
alpha	F7-C4	0.20±0.09	0.24±0.09	-1.68	0.10
**alpha**	**F7-T8**	**0.23±0.11**	**0.32±0.13**	**-2.98**	**< 0.001**
**alpha**	**F7-P7**	**0.37±0.19**	**0.62±0.09**	**-6.23**	**< 0.001**
**alpha**	**F7-P3**	**0.18±0.07**	**0.50±0.09**	**-14.63**	**< 0.001**
**alpha**	**F7-Pz**	**0.23±0.10**	**0.33±0.08**	**-4.29**	**< 0.001**
**alpha**	**F7-P4**	**0.24±0.10**	**0.30±0.10**	**-2.55**	**0.01**
**alpha**	**F7-P8**	**0.22±0.09**	**0.36±0.13**	**-4.83**	**< 0.001**
**alpha**	**F7-O1**	**0.33±0.14**	**0.48±0.11**	**-4.58**	**< 0.001**
**alpha**	**F7-O2**	**0.28±0.13**	**0.39±0.12**	**-3.31**	**< 0.001**
alpha	F3-Fz	0.64±0.24	0.60±0.20	0.74	0.46
**alpha**	**F3-F4**	**0.64±0.12**	**0.33±0.17**	**7.98**	**< 0.001**

**alpha**	**F3-F8**	**0.67±0.15**	**0.24±0.10**	**12.82**	**< 0.001**
**alpha**	**F3-T7**	**0.35±0.17**	**0.52±0.10**	**-4.67**	**< 0.001**
alpha	F3-C3	0.74±0.13	0.72±0.11	0.70	0.49
**alpha**	**F3-Cz**	**0.50±0.13**	**0.59±0.17**	**-2.20**	**0.03**
**alpha**	**F3-C4**	**0.82±0.08**	**0.36±0.14**	**15.31**	**< 0.001**
**alpha**	**F3-T8**	**0.43±0.14**	**0.22±0.10**	**6.30**	**< 0.001**
alpha	F3-P7	0.39±0.17	0.41±0.09	0.68	0.50
alpha	F3-P3	0.58±0.17	0.54±0.12	1.15	0.26
**alpha**	**F3-Pz**	**0.61±0.15**	**0.49±0.11**	**3.44**	**< 0.001**
**alpha**	**F3-P4**	**0.82±0.10**	**0.35±0.11**	**16.79**	**< 0.001**
**alpha**	**F3-P8**	**0.62±0.14**	**0.24±0.09**	**12.00**	**< 0.001**
**alpha**	**F3-O1**	**0.56±0.18**	**0.35±0.10**	**5.52**	**< 0.001**
**alpha**	**F3-O2**	**0.68±0.23**	**0.29±0.08**	**8.13**	**< 0.001**
alpha	Fz-F4	0.61±0.16	0.60±0.17	0.25	0.80
**alpha**	**Fz-F8**	**0.49±0.19**	**0.29±0.11**	**5.60**	**< 0.001**
alpha	Fz-T7	0.28±0.10	0.31±0.11	-1.90	0.28
**alpha**	**Fz-C3**	**0.71±0.27**	**0.51±0.16**	**3.35**	**< 0.001**
alpha	Fz-Cz	0.74±0.10	0.76±0.13	0.57	0.57
**alpha**	**Fz-C4**	**0.64±0.24**	**0.51±0.15**	**2.48**	**0.02**
**alpha**	**Fz-T8**	**0.44±0.14**	**0.31±0.11**	**3.88**	**< 0.001**
alpha	Fz-P7	0.27±0.12	0.32±0.12	-1.29	0.20
**alpha**	**Fz-P3**	**0.57±0.15**	**0.41±0.14**	**4.11**	**< 0.001**
**alpha**	**Fz-Pz**	**0.78±0.19**	**0.50±0.15**	**5.94**	**< 0.001**
**alpha**	**Fz-P4**	**0.74±0.29**	**0.41±0.15**	**5.31**	**< 0.001**
**alpha**	**Fz-P8**	**0.50±0.15**	**0.32±0.12**	**5.50**	**< 0.001**
**alpha**	**Fz-O1**	**0.45±0.21**	**0.32±0.13**	**2.76**	**0.01**
**alpha**	**Fz-O2**	**0.56±0.14**	**0.32±0.12**	**6.61**	**< 0.001**
**alpha**	**F4-F8**	**0.35±0.14**	**0.52±0.14**	**-4.42**	**< 0.001**
**alpha**	**F4-T7**	**0.46±0.14**	**0.24±0.11**	**6.57**	**< 0.001**
**alpha**	**F4-C3**	**0.61±0.18**	**0.39±0.16**	**4.96**	**< 0.001**
**alpha**	**F4-Cz**	**0.78±0.12**	**0.60±0.15**	**5.12**	**< 0.001**
**alpha**	**F4-C4**	**0.85±0.14**	**0.70±0.11**	**4.22**	**< 0.001**
**alpha**	**F4-T8**	**0.76±0.09**	**0.49±0.14**	**8.78**	**< 0.001**
**alpha**	**F4-P7**	**0.54±0.11**	**0.25±0.12**	**9.42**	**< 0.001**
**alpha**	**F4-P3**	**0.86±0.11**	**0.37±0.14**	**14.79**	**< 0.001**
**alpha**	**F4-Pz**	**0.76±0.11**	**0.52±0.14**	**7.30**	**< 0.001**
**alpha**	**F4-P4**	**0.81±0.06**	**0.52±0.13**	**11.17**	**< 0.001**
**alpha**	**F4-P8**	**0.79±0.15**	**0.41±0.14**	**9.81**	**< 0.001**
**alpha**	**F4-O1**	**0.46±0.16**	**0.29±0.13**	**4.39**	**< 0.001**
**alpha**	**F4-O2**	**0.77±0.09**	**0.36±0.14**	**13.29**	**< 0.001**

alpha	F8-T7	0.36±0.27	0.32±0.13	0.70	0.49
**alpha**	**F8-C3**	**0.69±0.16**	**0.27±0.10**	**11.75**	**< 0.001**
**alpha**	**F8-Cz**	**0.35±0.14**	**0.27±0.10**	**2.25**	**0.03**
alpha	F8-C4	0.46±0.17	0.52±0.08	-1.75	0.09
**alpha**	**F8-T8**	**0.30±0.13**	**0.78±0.08**	**-16.43**	**< 0.001**
**alpha**	**F8-P7**	**0.25±0.10**	**0.36±0.12**	**-3.70**	**< 0.001**
alpha	F8-P3	0.40±0.23	0.32±0.09	1.78	0.08
**alpha**	**F8-Pz**	**0.45±0.16**	**0.34±0.08**	**3.80**	**< 0.001**
alpha	F8-P4	0.51±0.11	0.48±0.08	1.30	0.31
**alpha**	**F8-P8**	**0.37±0.22**	**0.60±0.09**	**-4.84**	**< 0.001**
alpha	F8-O1	0.38±0.15	0.38±0.11	0.10	0.92
**alpha**	**F8-O2**	**0.33±0.14**	**0.47±0.10**	**-4.12**	**< 0.001**
**alpha**	**T7-C3**	**0.28±0.09**	**0.72±0.08**	**-19.26**	**< 0.001**
alpha	T7-Cz	0.40±0.13	0.39±0.11	0.52	0.60
alpha	T7-C4	0.39±0.10	0.38±0.10	0.35	0.73
**alpha**	**T7-T8**	**0.64±0.24**	**0.49±0.12**	**2.99**	**< 0.001**
**alpha**	**T7-P7**	**0.56±0.24**	**0.88±0.04**	**-6.93**	**< 0.001**
**alpha**	**T7-P3**	**0.55±0.12**	**0.76±0.09**	**-7.37**	**< 0.001**
**alpha**	**T7-Pz**	**0.34±0.11**	**0.54±0.08**	**-7.71**	**< 0.001**
**alpha**	**T7-P4**	**0.32±0.10**	**0.51±0.11**	**-6.51**	**< 0.001**
alpha	T7-P8	0.58±0.14	0.56±0.11	0.60	0.55
**alpha**	**T7-O1**	**0.39±0.19**	**0.74±0.08**	**-9.11**	**< 0.001**
**alpha**	**T7-O2**	**0.31±0.16**	**0.61±0.09**	**-8.49**	**< 0.001**
**alpha**	**C3-Cz**	**0.52±0.12**	**0.73±0.16**	**-5.55**	**< 0.001**
alpha	C3-C4	0.65±0.12	0.59±0.13	1.75	0.09
**alpha**	**C3-T8**	**0.54±0.22**	**0.41±0.11**	**2.68**	**0.01**
**alpha**	**C3-P7**	**0.34±0.20**	**0.71±0.08**	**-8.78**	**< 0.001**
**alpha**	**C3-P3**	**0.51±0.12**	**0.88±0.04**	**-15.21**	**< 0.001**
**alpha**	**C3-Pz**	**0.66±0.13**	**0.80±0.07**	**-4.86**	**< 0.001**
**alpha**	**C3-P4**	**0.80±0.10**	**0.64±0.12**	**5.66**	**< 0.001**
**alpha**	**C3-P8**	**0.40±0.15**	**0.49±0.09**	**-2.63**	**0.01**
**alpha**	**C3-O1**	**0.34±0.16**	**0.67±0.09**	**-9.29**	**< 0.001**
alpha	C3-O2	0.53±0.15	0.57±0.09	-1.50	0.30
alpha	Cz-C4	0.67±0.18	0.74±0.15	-1.48	0.15
**alpha**	**Cz-T8**	**0.62±0.17**	**0.39±0.13**	**5.74**	**< 0.001**
alpha	Cz-P7	0.35±0.13	0.41±0.14	-1.64	0.11
**alpha**	**Cz-P3**	**0.83±0.12**	**0.65±0.16**	**4.75**	**< 0.001**
alpha	Cz-Pz	0.84±0.05	0.81±0.16	0.92	0.36
alpha	Cz-P4	0.66±0.15	0.66±0.16	0.80	0.94
**alpha**	**Cz-P8**	**0.64±0.15**	**0.43±0.14**	**5.48**	**< 0.001**

alpha	Cz-O1	0.41±0.20	0.46±0.16	-1.10	0.32
**alpha**	**Cz-O2**	**0.57±0.16**	**0.47±0.16**	**2.21**	**0.03**
**alpha**	**C4-T8**	**0.59±0.12**	**0.70±0.12**	**-3.59**	**< 0.001**
alpha	C4-P7	0.45±0.15	0.44±0.11	0.15	0.88
**alpha**	**C4-P3**	**0.76±0.10**	**0.62±0.12**	**4.92**	**< 0.001**
alpha	C4-Pz	0.73±0.15	0.79±0.10	-1.72	0.09
**alpha**	**C4-P4**	**0.90±0.07**	**0.84±0.11**	**2.41**	**0.02**
alpha	C4-P8	0.73±0.15	0.68±0.13	1.17	0.25
alpha	C4-O1	0.49±0.18	0.52±0.13	0.75	0.46
alpha	C4-O2	0.72±0.22	0.64±0.14	1.71	0.09
**alpha**	**T8-P7**	**0.66±0.13**	**0.55±0.13**	**3.18**	**< 0.001**
**alpha**	**T8-P3**	**0.76±0.21**	**0.52±0.09**	**5.28**	**< 0.001**
alpha	T8-Pz	0.55±0.11	0.55±0.09	0.16	0.87
**alpha**	**T8-P4**	**0.57±0.13**	**0.74±0.11**	**-5.25**	**< 0.001**
**alpha**	**T8-P8**	**0.65±0.22**	**0.87±0.05**	**-5.90**	**< 0.001**
**alpha**	**T8-O1**	**0.33±0.18**	**0.59±0.11**	**-6.61**	**< 0.001**
**alpha**	**T8-O2**	**0.53±0.17**	**0.72±0.08**	**-5.31**	**< 0.001**
**alpha**	**P7-P3**	**0.44±0.16**	**0.84±0.06**	**-12.42**	**< 0.001**
**alpha**	**P7-Pz**	**0.30±0.11**	**0.62±0.09**	**-11.61**	**< 0.001**
**alpha**	**P7-P4**	**0.40±0.12**	**0.61±0.12**	**-6.38**	**< 0.001**
alpha	P7-P8	0.63±0.26	0.67±0.10	0.78	0.44
**alpha**	**P7-O1**	**0.58±0.26**	**0.91±0.04**	**-6.57**	**< 0.001**
**alpha**	**P7-O2**	**0.60±0.16**	**0.75±0.08**	**-4.35**	**< 0.001**
**alpha**	**P3-Pz**	**0.76±0.09**	**0.86±0.05**	**-5.38**	**< 0.001**
**alpha**	**P3-P4**	**0.67±0.11**	**0.75±0.13**	**-2.48**	**0.02**
**alpha**	**P3-P8**	**0.78±0.11**	**0.64±0.08**	**5.34**	**< 0.001**
**alpha**	**P3-O1**	**0.41±0.17**	**0.86±0.05**	**-13.68**	**< 0.001**
**alpha**	**P3-O2**	**0.55±0.14**	**0.75±0.07**	**-6.61**	**< 0.001**
**alpha**	**Pz-P4**	**0.78±0.14**	**0.86±0.10**	**-2.49**	**0.02**
**alpha**	**Pz-P8**	**0.57±0.18**	**0.65±0.09**	**-2.10**	**0.04**
**alpha**	**Pz-O1**	**0.36±0.17**	**0.71±0.10**	**-9.33**	**< 0.001**
**alpha**	**Pz-O2**	**0.56±0.17**	**0.73±0.10**	**-4.52**	**< 0.001**
**alpha**	**P4-P8**	**0.60±0.15**	**0.84±0.13**	**-6.49**	**< 0.001**
**alpha**	**P4-O1**	**0.44±0.17**	**0.71±0.14**	**-6.66**	**< 0.001**
**alpha**	**P4-O2**	**0.69±0.15**	**0.85±0.14**	**-4.30**	**< 0.001**
**alpha**	**P8-O1**	**0.69±0.12**	**0.75±0.09**	**-2.13**	**0.04**
**alpha**	**P8-O2**	**0.74±0.17**	**0.91±0.04**	**-5.11**	**< 0.001**
**alpha**	**O1-O2**	**0.67±0.22**	**0.86±0.06**	**-4.34**	**< 0.001**
beta	Fp1-Fp2	0.29±0.16	0.22±0.13	1.80	0.08
**beta**	**Fp1-F7**	**0.27±0.16**	**0.58±0.12**	**-8.11**	**< 0.001**

**beta**	**Fp1-F3**	**0.26±0.14**	**0.39±0.17**	**-3.34**	**< 0.001**
beta	Fp1-Fz	0.24±0.14	0.30±0.17	-1.50	0.14
beta	Fp1-F4	0.26±0.12	0.28±0.11	0.40	0.69
**beta**	**Fp1-F8**	**0.28±0.17**	**0.20±0.08**	**2.22**	**0.03**
**beta**	**Fp1-T7**	**0.58±0.14**	**0.37±0.13**	**5.83**	**< 0.001**
**beta**	**Fp1-C3**	**0.21±0.12**	**0.30±0.16**	**-2.45**	**0.02**
beta	Fp1-Cz	0.26±0.12	0.25±0.14	0.49	0.63
beta	Fp1-C4	0.24±0.12	0.22±0.10	0.82	0.41
**beta**	**Fp1-T8**	**0.34±0.13**	**0.19±0.09**	**4.76**	**< 0.001**
**beta**	**Fp1-P7**	**0.39±0.19**	**0.30±0.11**	**2.32**	**0.03**
beta	Fp1-P3	0.30±0.11	0.26±0.12	1.37	0.18
beta	Fp1-Pz	0.24±0.12	0.22±0.11	0.61	0.54
beta	Fp1-P4	0.23±0.12	0.21±0.09	0.65	0.52
**beta**	**Fp1-P8**	**0.33±0.17**	**0.21±0.08**	**3.45**	**< 0.001**
**beta**	**Fp1-O1**	**0.38±0.20**	**0.24±0.10**	**3.23**	**< 0.001**
beta	Fp1-O2	0.25±0.14	0.21±0.08	1.21	0.23
**beta**	**Fp2-F7**	**0.47±0.19**	**0.21±0.08**	**6.74**	**< 0.001**
**beta**	**Fp2-F3**	**0.59±0.23**	**0.27±0.09**	**6.82**	**< 0.001**
**beta**	**Fp2-Fz**	**0.51±0.17**	**0.28±0.14**	**5.60**	**< 0.001**
beta	Fp2-F4	0.40±0.17	0.37±0.14	0.85	0.40
beta	Fp2-F8	0.55±0.24	0.54±0.11	0.18	0.85
**beta**	**Fp2-T7**	**0.28±0.17**	**0.19±0.08**	**2.45**	**0.02**
**beta**	**Fp2-C3**	**0.54±0.16**	**0.21±0.09**	**9.45**	**< 0.001**
**beta**	**Fp2-Cz**	**0.37±0.19**	**0.23±0.11**	**3.42**	**< 0.001**
**beta**	**Fp2-C4**	**0.41±0.16**	**0.27±0.12**	**3.58**	**< 0.001**
beta	Fp2-T8	0.34±0.18	0.34±0.10	0.60	0.95
**beta**	**Fp2-P7**	**0.44±0.24**	**0.19±0.08**	**5.18**	**< 0.001**
**beta**	**Fp2-P3**	**0.31±0.16**	**0.20±0.09**	**3.46**	**< 0.001**
**beta**	**Fp2-Pz**	**0.36±0.16**	**0.21±0.09**	**4.52**	**< 0.001**
**beta**	**Fp2-P4**	**0.45±0.16**	**0.23±0.09**	**6.17**	**< 0.001**
**beta**	**Fp2-P8**	**0.40±0.21**	**0.26±0.09**	**3.23**	**< 0.001**
**beta**	**Fp2-O1**	**0.60±0.22**	**0.20±0.07**	**9.10**	**< 0.001**
**beta**	**Fp2-O2**	**0.60±0.16**	**0.22±0.07**	**11.75**	**< 0.001**
**beta**	**F7-F3**	**0.28±0.15**	**0.61±0.12**	**-9.19**	**< 0.001**
beta	F7-Fz	0.29±0.15	0.31±0.16	0.47	0.64
beta	F7-F4	0.23±0.13	0.26±0.11	0.89	0.38
**beta**	**F7-F8**	**0.49±0.23**	**0.21±0.09**	**5.99**	**< 0.001**
**beta**	**F7-T7**	**0.24±0.15**	**0.67±0.13**	**-11.23**	**< 0.001**
**beta**	**F7-C3**	**0.35±0.15**	**0.55±0.11**	**-5.64**	**< 0.001**
**beta**	**F7-Cz**	**0.22±0.13**	**0.32±0.14**	**-2.58**	**0.01**

beta	F7-C4	0.22±0.12	0.26±0.11	-1.23	0.22
beta	F7-T8	0.25±0.14	0.26±0.11	0.49	0.62
**beta**	**F7-P7**	**0.38±0.18**	**0.56±0.11**	**-4.31**	**< 0.001**
**beta**	**F7-P3**	**0.20±0.12**	**0.48±0.10**	**-9.16**	**< 0.001**
**beta**	**F7-Pz**	**0.24±0.13**	**0.37±0.11**	**-4.60**	**< 0.001**
**beta**	**F7-P4**	**0.25±0.12**	**0.31±0.10**	**-2.17**	**0.03**
**beta**	**F7-P8**	**0.24±0.14**	**0.32±0.09**	**-2.39**	**0.02**
**beta**	**F7-O1**	**0.32±0.19**	**0.45±0.09**	**-3.16**	**< 0.001**
**beta**	**F7-O2**	**0.27±0.16**	**0.37±0.08**	**-2.77**	**0.01**
beta	F3-Fz	0.62±0.22	0.62±0.19	0.16	0.87
**beta**	**F3-F4**	**0.61±0.11**	**0.34±0.17**	**7.11**	**< 0.001**
**beta**	**F3-F8**	**0.57±0.19**	**0.23±0.09**	**8.48**	**< 0.001**
**beta**	**F3-T7**	**0.34±0.19**	**0.50±0.13**	**-3.68**	**< 0.001**
beta	F3-C3	0.72±0.15	0.68±0.12	1.80	0.28
beta	F3-Cz	0.48±0.10	0.54±0.19	-1.50	0.14
**beta**	**F3-C4**	**0.77±0.11**	**0.35±0.16**	**11.84**	**< 0.001**
**beta**	**F3-T8**	**0.42±0.16**	**0.24±0.12**	**4.92**	**< 0.001**
beta	F3-P7	0.38±0.18	0.42±0.13	-1.20	0.31
beta	F3-P3	0.57±0.20	0.51±0.13	1.43	0.16
**beta**	**F3-Pz**	**0.60±0.11**	**0.45±0.15**	**4.27**	**< 0.001**
**beta**	**F3-P4**	**0.78±0.10**	**0.36±0.13**	**13.74**	**< 0.001**
**beta**	**F3-P8**	**0.61±0.14**	**0.27±0.10**	**10.20**	**< 0.001**
**beta**	**F3-O1**	**0.58±0.15**	**0.36±0.12**	**5.96**	**< 0.001**
**beta**	**F3-O2**	**0.69±0.19**	**0.30±0.10**	**9.64**	**< 0.001**
beta	Fz-F4	0.60±0.16	0.60±0.16	0.17	0.87
**beta**	**Fz-F8**	**0.43±0.17**	**0.30±0.12**	**3.28**	**< 0.001**
beta	Fz-T7	0.29±0.15	0.33±0.15	0.87	0.39
beta	Fz-C3	0.66±0.27	0.56±0.16	1.67	0.10
beta	Fz-Cz	0.71±0.11	0.75±0.13	-1.33	0.19
beta	Fz-C4	0.64±0.24	0.55±0.15	1.72	0.09
**beta**	**Fz-T8**	**0.42±0.16**	**0.29±0.14**	**3.21**	**< 0.001**
beta	Fz-P7	0.32±0.16	0.31±0.13	0.80	0.94
**beta**	**Fz-P3**	**0.55±0.14**	**0.45±0.16**	**2.50**	**0.02**
**beta**	**Fz-Pz**	**0.75±0.19**	**0.52±0.16**	**4.83**	**< 0.001**
**beta**	**Fz-P4**	**0.74±0.28**	**0.45±0.14**	**4.87**	**< 0.001**
**beta**	**Fz-P8**	**0.52±0.15**	**0.30±0.11**	**6.34**	**< 0.001**
**beta**	**Fz-O1**	**0.47±0.20**	**0.31±0.13**	**3.53**	**< 0.001**
**beta**	**Fz-O2**	**0.58±0.13**	**0.31±0.11**	**8.40**	**< 0.001**
**beta**	**F4-F8**	**0.34±0.17**	**0.56±0.12**	**-5.45**	**< 0.001**
**beta**	**F4-T7**	**0.44±0.15**	**0.27±0.13**	**4.60**	**< 0.001**

**beta**	**F4-C3**	**0.59±0.19**	**0.36±0.17**	**4.67**	**< 0.001**
**beta**	**F4-Cz**	**0.78±0.11**	**0.54±0.16**	**6.48**	**< 0.001**
**beta**	**F4-C4**	**0.85±0.09**	**0.64±0.12**	**7.30**	**< 0.001**
**beta**	**F4-T8**	**0.74±0.12**	**0.46±0.12**	**8.75**	**< 0.001**
**beta**	**F4-P7**	**0.51±0.13**	**0.27±0.14**	**6.92**	**< 0.001**
**beta**	**F4-P3**	**0.86±0.10**	**0.35±0.17**	**13.78**	**< 0.001**
**beta**	**F4-Pz**	**0.76±0.10**	**0.45±0.16**	**8.55**	**< 0.001**
**beta**	**F4-P4**	**0.80±0.06**	**0.48±0.13**	**12.42**	**< 0.001**
**beta**	**F4-P8**	**0.76±0.14**	**0.40±0.14**	**9.79**	**< 0.001**
**beta**	**F4-O1**	**0.47±0.16**	**0.29±0.14**	**4.44**	**< 0.001**
**beta**	**F4-O2**	**0.76±0.09**	**0.34±0.15**	**12.98**	**< 0.001**
beta	F8-T7	0.35±0.26	0.25±0.09	1.95	0.06
**beta**	**F8-C3**	**0.60±0.18**	**0.24±0.10**	**9.33**	**< 0.001**
beta	F8-Cz	0.33±0.15	0.30±0.10	0.82	0.42
**beta**	**F8-C4**	**0.42±0.18**	**0.52±0.09**	**-2.64**	**0.01**
**beta**	**F8-T8**	**0.27±0.17**	**0.64±0.12**	**-9.40**	**< 0.001**
beta	F8-P7	0.27±0.16	0.28±0.09	0.30	0.77
beta	F8-P3	0.37±0.23	0.28±0.09	1.79	0.08
beta	F8-Pz	0.40±0.17	0.34±0.09	1.79	0.08
beta	F8-P4	0.45±0.15	0.44±0.10	0.11	0.91
**beta**	**F8-P8**	**0.36±0.24**	**0.51±0.09**	**-3.10**	**< 0.001**
**beta**	**F8-O1**	**0.40±0.19**	**0.31±0.09**	**2.80**	**0.04**
beta	F8-O2	0.34±0.16	0.39±0.09	-1.38	0.18
**beta**	**T7-C3**	**0.28±0.15**	**0.67±0.13**	**-10.84**	**< 0.001**
beta	T7-Cz	0.37±0.15	0.41±0.14	-1.20	0.31
beta	T7-C4	0.39±0.13	0.35±0.13	1.15	0.26
**beta**	**T7-T8**	**0.57±0.22**	**0.36±0.11**	**4.58**	**< 0.001**
**beta**	**T7-P7**	**0.57±0.24**	**0.79±0.10**	**-4.49**	**< 0.001**
**beta**	**T7-P3**	**0.50±0.14**	**0.69±0.11**	**-5.74**	**< 0.001**
**beta**	**T7-Pz**	**0.35±0.13**	**0.52±0.11**	**-5.41**	**< 0.001**
**beta**	**T7-P4**	**0.34±0.14**	**0.44±0.10**	**-3.16**	**< 0.001**
**beta**	**T7-P8**	**0.56±0.16**	**0.44±0.10**	**3.45**	**< 0.001**
**beta**	**T7-O1**	**0.44±0.21**	**0.65±0.10**	**-4.87**	**< 0.001**
**beta**	**T7-O2**	**0.35±0.16**	**0.51±0.08**	**-4.79**	**< 0.001**
**beta**	**C3-Cz**	**0.49±0.13**	**0.71±0.15**	**-5.98**	**< 0.001**
**beta**	**C3-C4**	**0.62±0.12**	**0.51±0.13**	**3.20**	**< 0.001**
**beta**	**C3-T8**	**0.48±0.24**	**0.35±0.11**	**2.64**	**0.01**
**beta**	**C3-P7**	**0.36±0.19**	**0.68±0.09**	**-7.83**	**< 0.001**
**beta**	**C3-P3**	**0.48±0.14**	**0.85±0.04**	**-12.78**	**< 0.001**
**beta**	**C3-Pz**	**0.63±0.14**	**0.74±0.10**	**-3.56**	**< 0.001**

**beta**	**C3-P4**	**0.76±0.14**	**0.57±0.11**	**5.79**	**< 0.001**
beta	C3-P8	0.41±0.15	0.42±0.10	0.32	0.75
**beta**	**C3-O1**	**0.38±0.17**	**0.63±0.10**	**-6.60**	**< 0.001**
beta	C3-O2	0.56±0.15	0.52±0.09	1.51	0.14
beta	Cz-C4	0.68±0.15	0.71±0.14	0.80	0.43
**beta**	**Cz-T8**	**0.59±0.17**	**0.39±0.13**	**4.80**	**< 0.001**
**beta**	**Cz-P7**	**0.36±0.15**	**0.44±0.13**	**-2.21**	**0.03**
**beta**	**Cz-P3**	**0.80±0.13**	**0.65±0.14**	**4.80**	**< 0.001**
beta	Cz-Pz	0.81±0.08	0.78±0.15	0.76	0.45
beta	Cz-P4	0.65±0.13	0.66±0.13	0.18	0.86
**beta**	**Cz-P8**	**0.62±0.13**	**0.44±0.11**	**5.68**	**< 0.001**
beta	Cz-O1	0.41±0.20	0.47±0.13	-1.22	0.23
**beta**	**Cz-O2**	**0.57±0.14**	**0.47±0.12**	**2.77**	**0.01**
beta	C4-T8	0.59±0.11	0.63±0.15	-1.31	0.20
beta	C4-P7	0.44±0.13	0.40±0.12	1.25	0.22
**beta**	**C4-P3**	**0.77±0.09**	**0.54±0.12**	**8.50**	**< 0.001**
beta	C4-Pz	0.76±0.08	0.73±0.11	1.10	0.28
**beta**	**C4-P4**	**0.88±0.07**	**0.81±0.09**	**3.27**	**< 0.001**
beta	C4-P8	0.71±0.12	0.65±0.11	1.89	0.06
beta	C4-O1	0.48±0.16	0.46±0.12	0.55	0.59
**beta**	**C4-O2**	**0.73±0.16**	**0.59±0.12**	**3.68**	**< 0.001**
**beta**	**T8-P7**	**0.64±0.12**	**0.41±0.11**	**7.60**	**< 0.001**
**beta**	**T8-P3**	**0.72±0.19**	**0.42±0.09**	**7.56**	**< 0.001**
beta	T8-Pz	0.54±0.13	0.50±0.11	1.14	0.26
**beta**	**T8-P4**	**0.56±0.14**	**0.65±0.13**	**-2.65**	**0.01**
**beta**	**T8-P8**	**0.66±0.21**	**0.77±0.12**	**-2.35**	**0.02**
beta	T8-O1	0.39±0.18	0.46±0.11	-1.76	0.09
beta	T8-O2	0.56±0.17	0.61±0.11	-1.26	0.21
**beta**	**P7-P3**	**0.44±0.14**	**0.81±0.06**	**-12.61**	**< 0.001**
**beta**	**P7-Pz**	**0.34±0.14**	**0.62±0.09**	**-9.40**	**< 0.001**
**beta**	**P7-P4**	**0.41±0.13**	**0.53±0.10**	**-3.88**	**< 0.001**
beta	P7-P8	0.62±0.24	0.53±0.09	1.70	0.10
**beta**	**P7-O1**	**0.58±0.24**	**0.86±0.06**	**-5.75**	**< 0.001**
**beta**	**P7-O2**	**0.57±0.16**	**0.65±0.06**	**-2.26**	**0.03**
**beta**	**P3-Pz**	**0.73±0.12**	**0.84±0.06**	**-4.44**	**< 0.001**
beta	P3-P4	0.67±0.10	0.67±0.09	0.12	0.91
**beta**	**P3-P8**	**0.76±0.10**	**0.54±0.07**	**9.65**	**< 0.001**
**beta**	**P3-O1**	**0.43±0.16**	**0.81±0.08**	**-11.26**	**< 0.001**
**beta**	**P3-O2**	**0.57±0.12**	**0.67±0.06**	**-3.87**	**< 0.001**
**beta**	**Pz-P4**	**0.79±0.08**	**0.84±0.09**	**-2.70**	**0.04**

beta	Pz-P8	0.56±0.16	0.63±0.09	-1.84	0.07
**beta**	**Pz-O1**	**0.37±0.17**	**0.70±0.10**	**-8.78**	**< 0.001**
**beta**	**Pz-O2**	**0.57±0.15**	**0.72±0.09**	**-4.57**	**< 0.001**
**beta**	**P4-P8**	**0.60±0.13**	**0.81±0.10**	**-6.46**	**< 0.001**
**beta**	**P4-O1**	**0.45±0.16**	**0.63±0.12**	**-4.94**	**< 0.001**
**beta**	**P4-O2**	**0.70±0.11**	**0.80±0.11**	**-3.48**	**< 0.001**
**beta**	**P8-O1**	**0.69±0.13**	**0.62±0.10**	**2.42**	**0.02**
**beta**	**P8-O2**	**0.73±0.18**	**0.86±0.04**	**-3.57**	**< 0.001**
**beta**	**O1-O2**	**0.67±0.20**	**0.78±0.10**	**-2.66**	**0.01**
gamma	Fp1-Fp2	0.33±0.21	0.25±0.15	1.58	0.12
**gamma**	**Fp1-F7**	**0.29±0.22**	**0.51±0.16**	**-4.34**	**< 0.001**
gamma	Fp1-F3	0.30±0.19	0.36±0.20	-1.33	0.19
gamma	Fp1-Fz	0.29±0.20	0.34±0.21	0.88	0.38
gamma	Fp1-F4	0.31±0.20	0.30±0.15	0.11	0.92
gamma	Fp1-F8	0.31±0.21	0.24±0.13	1.42	0.16
**gamma**	**Fp1-T7**	**0.50±0.19**	**0.30±0.19**	**4.40**	**< 0.001**
gamma	Fp1-C3	0.26±0.19	0.32±0.20	-1.22	0.23
gamma	Fp1-Cz	0.30±0.20	0.31±0.17	0.14	0.89
gamma	Fp1-C4	0.30±0.19	0.30±0.16	0.10	0.99
gamma	Fp1-T8	0.31±0.21	0.26±0.15	1.60	0.29
gamma	Fp1-P7	0.38±0.22	0.28±0.17	1.84	0.07
gamma	Fp1-P3	0.30±0.20	0.30±0.17	0.10	0.99
gamma	Fp1-Pz	0.29±0.20	0.30±0.15	0.16	0.88
gamma	Fp1-P4	0.30±0.19	0.29±0.15	0.20	0.98
gamma	Fp1-P8	0.32±0.21	0.27±0.14	1.20	0.23
**gamma**	**Fp1-O1**	**0.38±0.22**	**0.27±0.15**	**2.14**	**0.04**
gamma	Fp1-O2	0.32±0.20	0.27±0.13	1.50	0.30
**gamma**	**Fp2-F7**	**0.46±0.20**	**0.24±0.13**	**5.10**	**< 0.001**
**gamma**	**Fp2-F3**	**0.65±0.24**	**0.29±0.14**	**6.97**	**< 0.001**
**gamma**	**Fp2-Fz**	**0.62±0.17**	**0.31±0.18**	**6.68**	**< 0.001**
**gamma**	**Fp2-F4**	**0.57±0.19**	**0.35±0.18**	**4.42**	**< 0.001**
gamma	Fp2-F8	0.56±0.27	0.47±0.13	1.52	0.14
**gamma**	**Fp2-T7**	**0.37±0.25**	**0.22±0.12**	**2.77**	**0.01**
**gamma**	**Fp2-C3**	**0.62±0.18**	**0.28±0.14**	**8.11**	**< 0.001**
**gamma**	**Fp2-Cz**	**0.52±0.20**	**0.27±0.16**	**5.24**	**< 0.001**
**gamma**	**Fp2-C4**	**0.56±0.20**	**0.30±0.16**	**5.36**	**< 0.001**
**gamma**	**Fp2-T8**	**0.47±0.24**	**0.28±0.15**	**3.50**	**< 0.001**
**gamma**	**Fp2-P7**	**0.55±0.24**	**0.24±0.13**	**6.00**	**< 0.001**
**gamma**	**Fp2-P3**	**0.48±0.22**	**0.26±0.13**	**4.53**	**< 0.001**
**gamma**	**Fp2-Pz**	**0.52±0.20**	**0.26±0.13**	**5.66**	**< 0.001**

**gamma**	**Fp2-P4**	**0.59±0.18**	**0.27±0.14**	**7.18**	**< 0.001**
**gamma**	**Fp2-P8**	**0.55±0.23**	**0.26±0.13**	**5.86**	**< 0.001**
**gamma**	**Fp2-O1**	**0.68±0.22**	**0.23±0.12**	**9.27**	**< 0.001**
**gamma**	**Fp2-O2**	**0.68±0.15**	**0.24±0.12**	**12.11**	**< 0.001**
**gamma**	**F7-F3**	**0.32±0.19**	**0.59±0.18**	**-5.73**	**< 0.001**
gamma	F7-Fz	0.32±0.19	0.42±0.21	-1.96	0.05
gamma	F7-F4	0.31±0.18	0.32±0.19	0.12	0.91
**gamma**	**F7-F8**	**0.47±0.23**	**0.25±0.15**	**4.12**	**< 0.001**
**gamma**	**F7-T7**	**0.29±0.20**	**0.55±0.19**	**-5.30**	**< 0.001**
**gamma**	**F7-C3**	**0.34±0.18**	**0.54±0.18**	**-4.21**	**< 0.001**
**gamma**	**F7-Cz**	**0.29±0.19**	**0.41±0.20**	**-2.36**	**0.02**
gamma	F7-C4	0.30±0.18	0.35±0.19	-1.30	0.31
gamma	F7-T8	0.30±0.19	0.31±0.19	0.27	0.78
gamma	F7-P7	0.37±0.19	0.47±0.19	-1.86	0.07
**gamma**	**F7-P3**	**0.28±0.18**	**0.49±0.17**	**-4.31**	**< 0.001**
**gamma**	**F7-Pz**	**0.29±0.19**	**0.43±0.17**	**-2.82**	**0.01**
gamma	F7-P4	0.31±0.18	0.38±0.17	-1.55	0.13
gamma	F7-P8	0.30±0.19	0.34±0.17	0.83	0.41
gamma	F7-O1	0.35±0.22	0.41±0.17	-1.11	0.27
gamma	F7-O2	0.33±0.19	0.37±0.16	0.95	0.35
gamma	F3-Fz	0.72±0.21	0.68±0.20	0.69	0.49
**gamma**	**F3-F4**	**0.70±0.16**	**0.46±0.22**	**4.82**	**< 0.001**
**gamma**	**F3-F8**	**0.54±0.25**	**0.30±0.18**	**4.12**	**< 0.001**
gamma	F3-T7	0.40±0.24	0.52±0.21	-2.00	0.05
gamma	F3-C3	0.71±0.20	0.72±0.15	0.17	0.87
gamma	F3-Cz	0.63±0.16	0.62±0.21	0.28	0.78
**gamma**	**F3-C4**	**0.80±0.12**	**0.51±0.21**	**6.50**	**< 0.001**
gamma	F3-T8	0.50±0.25	0.38±0.21	1.93	0.06
gamma	F3-P7	0.50±0.24	0.52±0.19	0.25	0.81
gamma	F3-P3	0.68±0.19	0.61±0.18	1.51	0.14
**gamma**	**F3-Pz**	**0.69±0.16**	**0.58±0.17**	**2.62**	**0.01**
**gamma**	**F3-P4**	**0.80±0.14**	**0.52±0.18**	**6.63**	**< 0.001**
**gamma**	**F3-P8**	**0.70±0.17**	**0.45±0.19**	**5.30**	**< 0.001**
**gamma**	**F3-O1**	**0.67±0.19**	**0.49±0.19**	**3.64**	**< 0.001**
**gamma**	**F3-O2**	**0.73±0.21**	**0.47±0.17**	**5.13**	**< 0.001**
gamma	Fz-F4	0.72±0.15	0.66±0.18	1.38	0.17
gamma	Fz-F8	0.49±0.24	0.41±0.17	1.36	0.18
gamma	Fz-T7	0.40±0.23	0.48±0.21	-1.43	0.16
gamma	Fz-C3	0.71±0.22	0.71±0.17	0.10	0.99
gamma	Fz-Cz	0.76±0.13	0.80±0.13	-1.12	0.27

gamma	Fz-C4	0.74±0.19	0.68±0.18	1.18	0.24
gamma	Fz-T8	0.53±0.23	0.47±0.21	0.98	0.33
gamma	Fz-P7	0.49±0.21	0.52±0.19	0.45	0.65
gamma	Fz-P3	0.65±0.17	0.64±0.18	0.20	0.84
**gamma**	**Fz-Pz**	**0.79±0.16**	**0.67±0.18**	**2.50**	**0.02**
**gamma**	**Fz-P4**	**0.80±0.20**	**0.63±0.18**	**3.35**	**< 0.001**
**gamma**	**Fz-P8**	**0.66±0.16**	**0.53±0.19**	**2.86**	**0.01**
**gamma**	**Fz-O1**	**0.63±0.17**	**0.52±0.19**	**2.32**	**0.02**
**gamma**	**Fz-O2**	**0.70±0.15**	**0.53±0.18**	**4.40**	**< 0.001**
**gamma**	**F4-F8**	**0.42±0.25**	**0.56±0.15**	**-2.59**	**0.01**
gamma	F4-T7	0.48±0.22	0.37±0.19	1.95	0.06
**gamma**	**F4-C3**	**0.66±0.21**	**0.52±0.20**	**2.62**	**0.01**
**gamma**	**F4-Cz**	**0.84±0.09**	**0.61±0.20**	**5.69**	**< 0.001**
**gamma**	**F4-C4**	**0.88±0.08**	**0.68±0.17**	**5.74**	**< 0.001**
**gamma**	**F4-T8**	**0.71±0.18**	**0.49±0.19**	**4.52**	**< 0.001**
**gamma**	**F4-P7**	**0.61±0.19**	**0.42±0.19**	**3.74**	**< 0.001**
**gamma**	**F4-P3**	**0.87±0.09**	**0.51±0.19**	**9.26**	**< 0.001**
**gamma**	**F4-Pz**	**0.81±0.11**	**0.57±0.18**	**6.20**	**< 0.001**
**gamma**	**F4-P4**	**0.85±0.07**	**0.58±0.16**	**8.32**	**< 0.001**
**gamma**	**F4-P8**	**0.82±0.14**	**0.51±0.18**	**6.93**	**< 0.001**
**gamma**	**F4-O1**	**0.63±0.19**	**0.44±0.20**	**3.64**	**< 0.001**
**gamma**	**F4-O2**	**0.82±0.10**	**0.48±0.19**	**8.62**	**< 0.001**
gamma	F8-T7	0.39±0.26	0.29±0.14	1.79	0.08
**gamma**	**F8-C3**	**0.55±0.26**	**0.34±0.15**	**3.61**	**< 0.001**
gamma	F8-Cz	0.41±0.24	0.40±0.15	0.28	0.78
gamma	F8-C4	0.45±0.24	0.54±0.14	-1.67	0.10
**gamma**	**F8-T8**	**0.35±0.25**	**0.53±0.15**	**-3.23**	**< 0.001**
gamma	F8-P7	0.36±0.24	0.33±0.14	0.49	0.62
gamma	F8-P3	0.42±0.25	0.37±0.14	0.92	0.36
gamma	F8-Pz	0.45±0.24	0.41±0.13	0.81	0.42
gamma	F8-P4	0.47±0.24	0.47±0.14	0.30	0.97
gamma	F8-P8	0.44±0.26	0.45±0.13	0.21	0.83
**gamma**	**F8-O1**	**0.49±0.23**	**0.35±0.13**	**2.73**	**0.01**
gamma	F8-O2	0.42±0.25	0.39±0.14	0.52	0.61
**gamma**	**T7-C3**	**0.33±0.23**	**0.64±0.21**	**-5.12**	**< 0.001**
gamma	T7-Cz	0.45±0.21	0.53±0.21	-1.41	0.16
gamma	T7-C4	0.44±0.22	0.49±0.18	0.83	0.41
**gamma**	**T7-T8**	**0.55±0.26**	**0.41±0.18**	**2.37**	**0.02**
gamma	T7-P7	0.58±0.25	0.69±0.19	-1.79	0.08
**gamma**	**T7-P3**	**0.51±0.21**	**0.67±0.18**	**-2.95**	**< 0.001**

**gamma**	**T7-Pz**	**0.42±0.22**	**0.59±0.18**	**-3.80**	**< 0.001**
**gamma**	**T7-P4**	**0.42±0.22**	**0.53±0.17**	**-2.25**	**0.03**
gamma	T7-P8	0.55±0.21	0.48±0.17	1.37	0.18
gamma	T7-O1	0.51±0.23	0.60±0.19	-1.57	0.12
gamma	T7-O2	0.43±0.23	0.52±0.16	-1.71	0.09
**gamma**	**C3-Cz**	**0.58±0.20**	**0.79±0.16**	**-4.47**	**< 0.001**
gamma	C3-C4	0.66±0.18	0.67±0.15	0.22	0.83
gamma	C3-T8	0.50±0.24	0.49±0.19	0.10	0.92
**gamma**	**C3-P7**	**0.44±0.22**	**0.72±0.13**	**-5.77**	**< 0.001**
**gamma**	**C3-P3**	**0.55±0.20**	**0.87±0.05**	**-7.92**	**< 0.001**
**gamma**	**C3-Pz**	**0.65±0.20**	**0.81±0.09**	**-3.87**	**< 0.001**
gamma	C3-P4	0.75±0.17	0.71±0.11	0.93	0.36
gamma	C3-P8	0.53±0.21	0.60±0.15	-1.45	0.15
**gamma**	**C3-O1**	**0.53±0.22**	**0.70±0.14**	**-3.47**	**< 0.001**
gamma	C3-O2	0.64±0.19	0.65±0.13	0.34	0.73
gamma	Cz-C4	0.78±0.12	0.78±0.16	0.50	0.96
gamma	Cz-T8	0.62±0.21	0.52±0.21	1.84	0.07
gamma	Cz-P7	0.51±0.20	0.61±0.18	-1.84	0.07
gamma	Cz-P3	0.83±0.11	0.77±0.15	1.80	0.08
gamma	Cz-Pz	0.84±0.08	0.83±0.15	0.41	0.68
gamma	Cz-P4	0.76±0.12	0.76±0.15	0.10	0.99
**gamma**	**Cz-P8**	**0.73±0.12**	**0.62±0.16**	**2.86**	**0.01**
gamma	Cz-O1	0.59±0.18	0.63±0.18	0.87	0.39
gamma	Cz-O2	0.70±0.13	0.64±0.17	1.57	0.12
gamma	C4-T8	0.61±0.21	0.63±0.21	0.27	0.79
gamma	C4-P7	0.55±0.21	0.57±0.17	0.28	0.78
**gamma**	**C4-P3**	**0.82±0.10**	**0.70±0.13**	**3.87**	**< 0.001**
gamma	C4-Pz	0.81±0.09	0.80±0.10	0.43	0.67
**gamma**	**C4-P4**	**0.90±0.07**	**0.85±0.07**	**2.81**	**0.01**
gamma	C4-P8	0.78±0.12	0.73±0.13	1.44	0.16
gamma	C4-O1	0.63±0.18	0.62±0.16	0.34	0.73
gamma	C4-O2	0.78±0.17	0.70±0.13	1.99	0.05
**gamma**	**T8-P7**	**0.65±0.17**	**0.47±0.18**	**3.86**	**< 0.001**
**gamma**	**T8-P3**	**0.70±0.24**	**0.53±0.18**	**3.70**	**< 0.001**
gamma	T8-Pz	0.57±0.22	0.58±0.18	0.13	0.90
gamma	T8-P4	0.59±0.21	0.64±0.20	-1.10	0.31
gamma	T8-P8	0.69±0.23	0.68±0.18	0.11	0.91
gamma	T8-O1	0.52±0.24	0.50±0.18	0.26	0.79
gamma	T8-O2	0.64±0.19	0.59±0.19	1.70	0.29
**gamma**	**P7-P3**	**0.56±0.21**	**0.84±0.08**	**-6.48**	**< 0.001**

**gamma**	**P7-Pz**	**0.49±0.20**	**0.72±0.12**	**-5.18**	**< 0.001**
**gamma**	**P7-P4**	**0.54±0.20**	**0.65±0.13**	**-2.62**	**0.01**
gamma	P7-P8	0.67±0.25	0.59±0.15	1.50	0.14
**gamma**	**P7-O1**	**0.66±0.25**	**0.84±0.13**	**-3.39**	**< 0.001**
gamma	P7-O2	0.66±0.18	0.68±0.13	0.49	0.62
**gamma**	**P3-Pz**	**0.77±0.15**	**0.88±0.06**	**-3.64**	**< 0.001**
gamma	P3-P4	0.75±0.12	0.78±0.08	-1.28	0.21
**gamma**	**P3-P8**	**0.82±0.09**	**0.68±0.12**	**4.93**	**< 0.001**
**gamma**	**P3-O1**	**0.60±0.17**	**0.83±0.14**	**-5.67**	**< 0.001**
**gamma**	**P3-O2**	**0.68±0.16**	**0.76±0.10**	**-2.22**	**0.03**
gamma	Pz-P4	0.83±0.10	0.88±0.07	-1.92	0.06
gamma	Pz-P8	0.68±0.16	0.74±0.10	-1.72	0.09
**gamma**	**Pz-O1**	**0.56±0.19**	**0.77±0.12**	**-4.99**	**< 0.001**
**gamma**	**Pz-O2**	**0.69±0.15**	**0.80±0.09**	**-3.37**	**< 0.001**
**gamma**	**P4-P8**	**0.72±0.15**	**0.85±0.08**	**-4.11**	**< 0.001**
**gamma**	**P4-O1**	**0.62±0.19**	**0.73±0.12**	**-2.50**	**0.02**
**gamma**	**P4-O2**	**0.78±0.14**	**0.84±0.08**	**-2.20**	**0.03**
**gamma**	**P8-O1**	**0.75±0.13**	**0.67±0.15**	**2.28**	**0.03**
gamma	P8-O2	0.78±0.19	0.85±0.09	-1.88	0.07
gamma	O1-O2	0.73±0.22	0.79±0.13	-1.43	0.16
